# Light‐controlled smart materials: Supramolecular regulation and applications

**DOI:** 10.1002/smo.20240036

**Published:** 2024-09-15

**Authors:** Zi‐Hao Liao, Feng Wang

**Affiliations:** ^1^ Key Laboratory of Materials Chemistry for Energy Conversion and Storage (Huazhong University of Science and Technology) of Ministry of Education Hubei Key Laboratory of Material Chemistry and Service Failure Hubei Engineering Research Center for Biomaterials and Medical Protective Materials School of Chemistry and Chemical Engineering Huazhong University of Science and Technology Wuhan China; ^2^ Guangdong Provincial Key Laboratory of Manufacturing Equipment Digitization Guangdong HUST Industrial Technology Research Institute Dongguan China

**Keywords:** intelligent materials, photochemistry, photochromism, photoresponsive materials, supramolecular chemistry

## Abstract

Smart materials serve as the fundamental cornerstone supporting humanity's transition into the intelligent era. Smart materials possess the capability to perceive external stimuli and respond accordingly. Light‐controlled smart materials (LCSMs) are a significant category that can sense and respond to light stimuli. Light, being a non‐invasive, precisely regulated, and remotely controllable source of physical stimulation, makes LCSMs indispensable in certain application scenarios. Recently, the construction of LCSMs using supramolecular strategies has emerged as a significant research focus. Supramolecular assembly, based on non‐covalent bonding, offers dynamic, reversible, and biomimetic properties. By integrating supramolecular systems with photoresponsive molecular building blocks, these materials can achieve synergistic and rich intelligent stimulus responses. This review delves into the latest research advancements in LCSMs based on supramolecular strategies. There are four sections in this review. The first section defines LCSMs and outlines their advantages. The second section discusses the design approaches of supramolecular LCSMs. The third section highlights the latest advancements on supramolecular LCSMs over the past 3 years. The fourth section summarizes the current research and provides insights into the future development of this field.

## Graphical abstract

Light‐controlled smart materials (LCSM) represent the material basis for human beings to enter the era of optoelectronic intelligence. Supramolecular strategies are used to construct LCSMs to make the materials with more synergistic and rich intelligent stimulus responses. This review focuses on recent research progresses on supramolecular LCSMs, especially focusing on the basic strategies of designing supramolecular LCSMs as well as various successful applications.

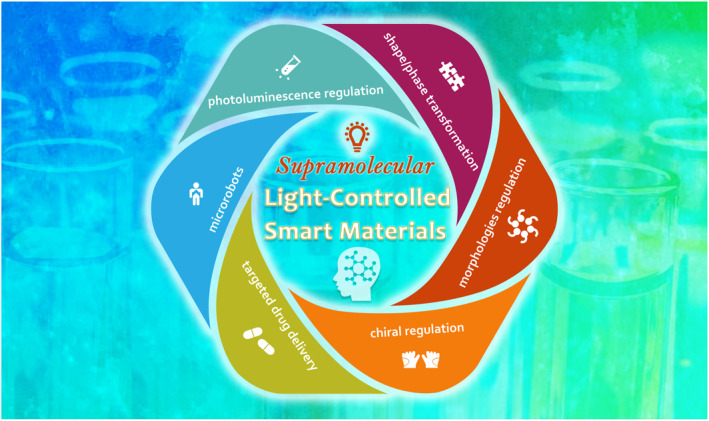

## 1 INTRODUCTION

**SCHEME 1:**
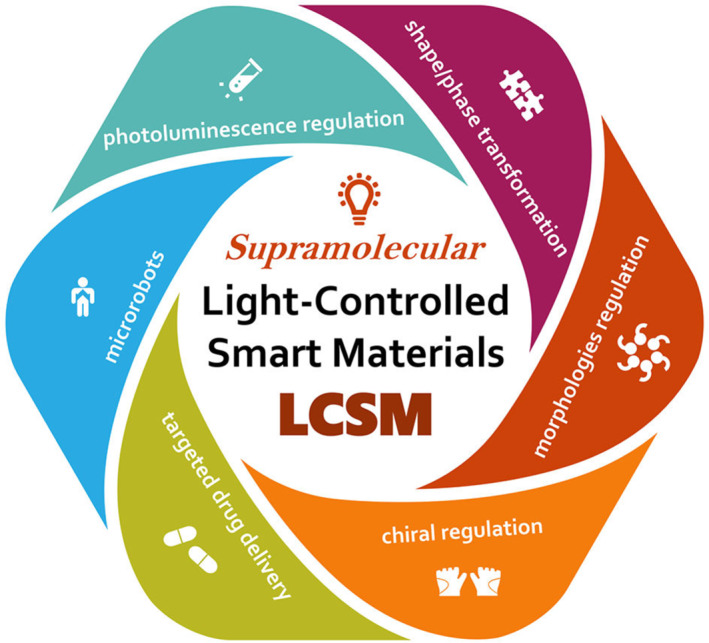
Light‐controlled smart materials (LCSMs) and their applications.

The development of human society is intrinsically linked to the use and advancement of materials.^[1]^ Historically, the utilization of materials by humans has evolved through three distinct stages: natural materials, synthetic polymer materials, and artificially designed functional materials.^[2]^ We have now entered an era characterized by intelligent materials. Intelligent materials, or smart materials, form the foundational basis for an all‐encompassing transition into the intelligent era.^[3]^ These materials possess the capability to perceive external stimuli and respond accordingly. Among these, light‐controlled smart materials (LCSM) are a significant category that can sense and respond to light stimuli (Scheme 1).

LCSMs offer several advantages over other stimulus‐responsive smart materials.[^4^, ^5^] Firstly, light is a non‐invasive physical stimulus that can activate and drive a closed system without introducing extraneous species such as chemicals. Secondly, light is a highly controllable stimulus. Its wavelength, intensity, and radiation range can be precisely controlled. LCSMs are sensitive to these parameters, particularly the wavelength, allowing for specific identification. The spot size can also be finely tuned to achieve localized stimulation of the material's micro‐regions, enhancing the precision of light manipulation. Thirdly, light as an exogenous stimulus can be operated remotely, overcoming spatial constraints between the stimulus source and the material. Consequently, LCSMs are often applied in specific scenarios. In the era of intelligent optoelectronics, the design and development of LCSMs have become a focal point of current research.[^6^, ^7^] Currently, LCSMs have already found successful applications in semiconductor industries, information storage and encryption, intelligent wearables, medical diagnostics and treatment, as well as sensing.[^8^, ^9^]

Given the surge of technologies such as artificial intelligence, humanoid robots, and brain‐computer interfaces, the demand for LCSMs is expected to increase in the future. At present, smarter, more complex, and more precise LCSMs are desired, and the application scenarios for LCSMs are expanding. Despite the existence of numerous comprehensive review articles on smart materials or photoresponsive materials, reviews specifically focusing on LCSM, particularly the review devoting to the LCSMs designed through supramolecular strategies, remain rare. This review aims to address this gap by concentrating on the research progress of LCSM based on supramolecular self‐assembly strategies.

The review is divided into four sections. The first section serves as an introduction, outlining the definition and advantages of LCSMs. The second section delves into the design strategies for these materials, emphasizing the supramolecular strategies in their construction. The third section highlights the research advancements in this field over the past 3 years, focusing on various applications of the materials: (i) light‐controlled luminescence regulation, (ii) light‐controlled macroscopic shape/phase transformation, (iii) light‐controlled microscopic morphology regulation, (iv) light‐controlled chiral regulation, (v) light‐controlled targeted drug delivery, and (vi) light‐controlled microrobots. The fourth section summarizes the current research and offers prospects for future development.

## 2 SUPRAMOLECULAR STRATEGIES TO LIGHT‐CONTROLLED SMART MATERIALS

**SCHEME 2:**
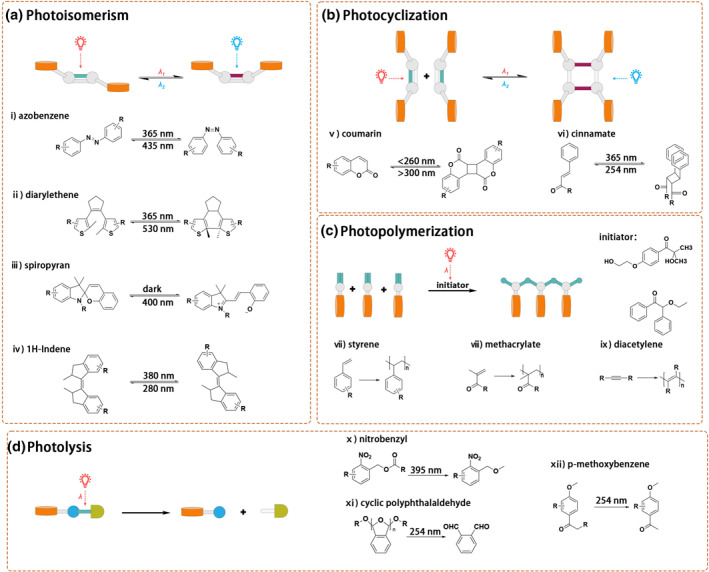
Photoresponsive molecular building blocks (PMBBs) used in supramolecular LCSMs are reviewed in this review.

The key point of the creation of LCSMs is to incorporate photoresponsive molecule building blocks (PMBBs) into the substrate system at the molecular level. PMBBs act as molecular triggers that can accurately identify light stimuli and elicit subsequent responses. From the perspective of light‐induced covalent bond change, there are four main mechanisms of PMBBs (Scheme 2), photoisomerism, photocyclization, photopolymerization, and photocleavage. The essence of these four mechanisms is the covalent bond isomerization (photoisomerism), formation (photocyclization and photopolymerization), and cleavage (photocleavage) of the PMBBs via intramolecular or intermolecular photoreactions. The former two types, photoisomerism and photocyclization, are often reversible reactions through irradiation under different lights. This makes the LCSMs containing these two PMBBs have reversible responses to light. Photopolymerization and photocleavage are irreversible reactions, making the LCSMs based on these PMBBs only have a one‐way response to light. Scheme 2 summarizes the chemical structures and photoreactions of the PMBBs used in the reviewed LCSMs. PMBBs, such as azobenzene,[^10^, ^11^] diarylethene,[^12^, ^13^] spiropyran,[^14^, ^15^] and coumarin, of photoisomeric properties are also referred to as molecular photoswitch. These molecules can be isomerized via the cis–trans isomerization of double bonds within the molecule or via a photoinduced ring closure/open reaction, leading to changes in their structural characteristics and photophysical properties. Molecules that can undergo photoinduced cyclization reactions include coumarin[^16^, ^17^] and cinnamate.^[18]^ Their ability to dimerize when exposed to ultraviolet light makes them attractive candidates for triggering controlled changes in polymer structure. Partially incorporating such molecules into polymer chains allows for the modulation of the self‐assembly mechanism, resulting in tuneable and reversible material properties.^[19]^ Photopolymerization refers to the polymerization of monomer molecules initiated by light, which can be directly triggered by photoexcitation or induced by a photoinitiator.^[20]^ Such monomers include styrene,^[21]^ diacetylene,^[22]^ and methacrylate.^[23]^ Molecules with photocleavage properties usually include nitrobenzyl, p‐methoxybenzene and cyclic polyphthalaldehyde.^[24]^ These structures are unstable under light irradiation, and incorporating such molecules into polymers enables direct light‐induced fracture of polymer assemblies.

Currently, the construction of LCSMs is progressing towards greater intelligence and complexity with supramolecular assembly demonstrating distinct advantages. Supramolecular assembly, featuring dynamics, reversibility, and biomimicry, leverages non‐covalent interactions to form assemblies with new functionalities.[^25^, ^26^] This strategy employs non‐covalent interaction forces such as electrostatic, hydrophilic and hydrophobic interactions, host‐guest interactions, and hydrogen bonding to form highly ordered and complex systems, circumventing lengthy and complex synthesis processes. The dynamic and reversible nature of non‐covalent bonds affords material systems based on supramolecular assembly with stimulus responses. When combined with PMBBs, the supramolecular assembly system often results in richer and more complex stimulus responses due to the synergistic effects not present in single systems.

**SCHEME 3:**
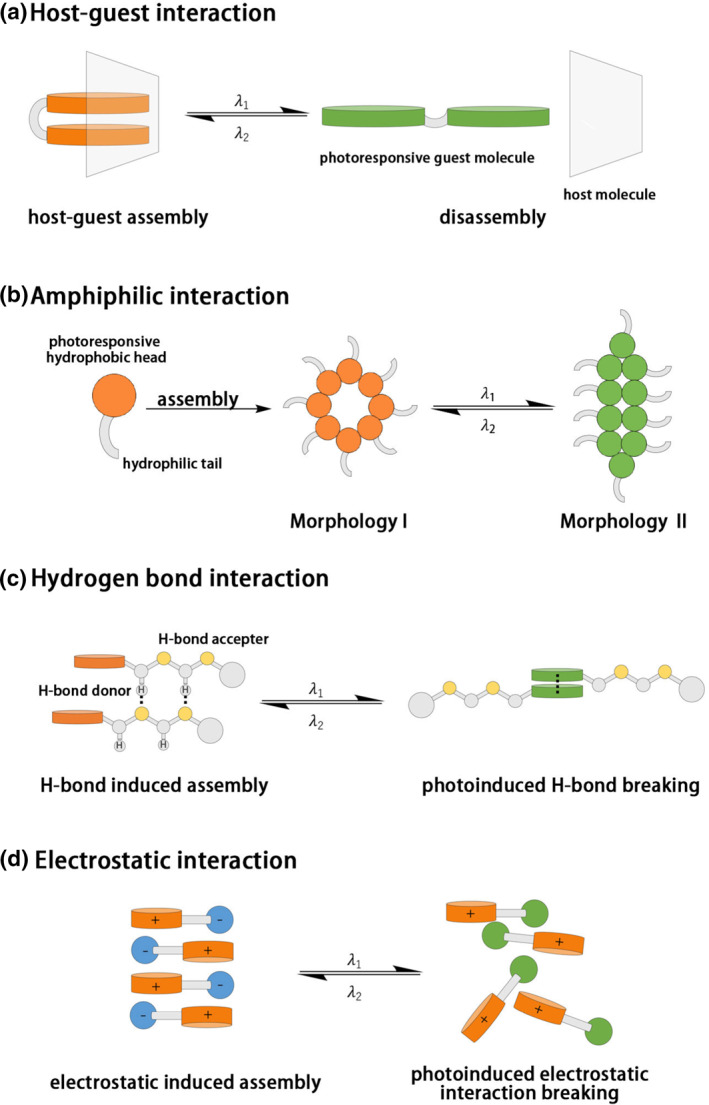
Four supramolecular assembly strategies in this review.

Four supramolecular assembly strategies are mainly used in the construction of LCSMs (Scheme 3). The first one is the host‐guest recognition between a PMBB and a macrocyclic compound. With the rapid development of macrocyclic chemistry, supramolecular host‐guest recognition systems incorporating photoresponsive molecules have become a research hotspot.[^27^, ^28^] Widely used macrocyclic compounds include cyclodextrins,[^29^, ^30^] cucurbit[n]urils,^[31]^ crown ethers,^[32]^ and pillararenes,^[33]^ which function as hosts to form host‐guest complexes with molecular photoswitch guest molecules, such as diarylethene and azobenzene. Through structural photoisomerization of these guest molecules, the assembly structure of the host‐guest complex can be either promoted or disrupted, leading to supramolecular optical switching or motor effects. The second strategy is amphiphilic supramolecular self‐assembly. Amphiphilic molecules or polymers can self‐assemble or disassemble in response to solvent environments. Incorporation of PMBB with amphiphilic molecules or polymers can make the amphiphilic self‐assembly with photoresponsive properties.[^34^, ^35^] Electrostatic interaction is the third strategy to construct supramolecular LCSMs. Molecules or polymers with charged groups often exhibit excellent water solubility and strong electrostatic interaction with the species with opposite charges. These properties are frequently explored as the basis of supramolecular hydrogels. By coupling PMBB with charged molecules or polymers, it becomes possible to use light to alter the charge density, regulate electrostatic interactions, and consequently modify the physical properties of supramolecular hydrogels.[^34^, ^36^] Hydrogen bond interaction is the fourth strategy. Hydrogen bond interaction is an ideal non‐covalent interaction for fabricating supramolecular materials due to its high selectivity, directionality, and dynamics. By integrating PMBB with hydrogen bond‐containing molecules/polymers, the intensity of hydrogen bond interactions can be controlled by light, thus enabling the creation of dynamic, light‐responsive hydrogen‐bond constructed materials.[^37^, ^38^]

**SCHEME 4:**
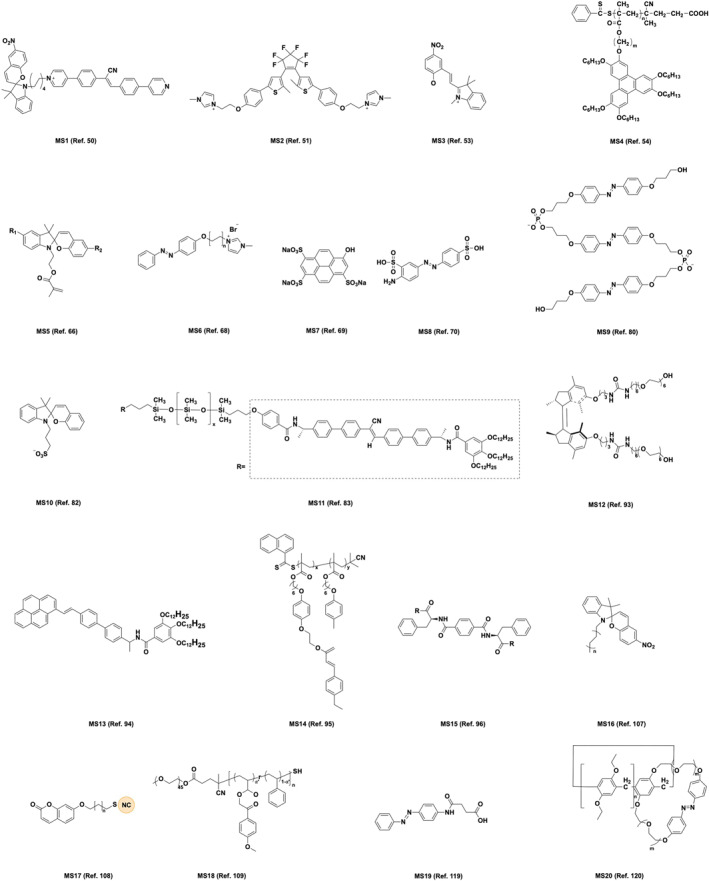
Molecular structures in the important research work of this review.

In addition, we summarize the molecular structures in the important research work of this review in Scheme 4.

## 3 SUPRAMOLECULAR LIGHT‐CONTROLLED SMART MATERIALS/SYSTEMS

### 3.1 Light‐controlled photoluminescence regulation

Photoluminescence (PL), including fluorescence and phosphoresce, is one of the typical luminescence phenomena. The changes in wavelength (discoloration) or intensity (on‐off switch) of PL are both easily observed by a humans' naked eye.[^39^, ^40^, ^41^] Accordingly, PL has extensive applications in biological imaging, medical diagnostics, display technologies, encryption, and anti‐counterfeiting.[^42^, ^43^, ^44^, ^45^] Combination of PL chromophores with supramolecular systems to fabricate light‐controlled smart luminescent materials indicates that PL behaviors can be regulated via light stimuli.[^46^, ^47^, ^48^, ^49^]

**FIGURE 1:**
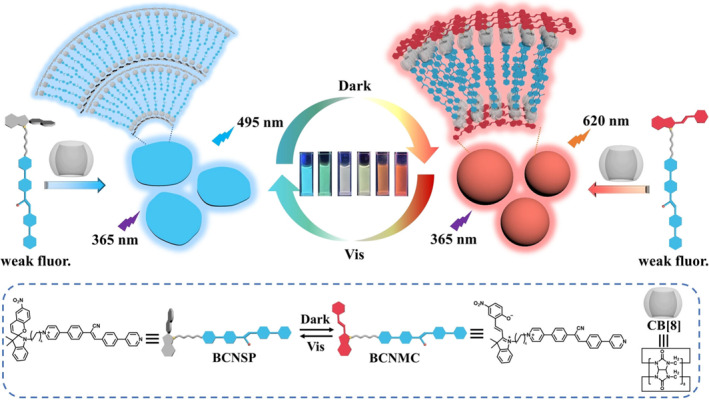
Schematic illustration of the construction and modulation of the multicolor, reversible supramolecular shuttle BCNMC⊂CB[8]. CB, cucurbit. *Source*: Copyright 2023, Wiley‐VCH GmbH.

The supramolecular host‐guest interaction between a photoresponsive guest molecule and a macrocyclic host molecule is one of the strategies to regulate PL colors via light stimuli. In this endeavor, Liu et al. employed host‐guest interaction to develop a light‐controlled multicolor fluorescence system for dynamic and reversible information storage (Figure 1).^[50]^ They encapsulated spiropyran‐modified cyanostilbene (BCNMC) (**MS1**) into macrocyclic in Ref. cucurbit[8]uril (CB[8]) to form a host‐guest complex. The blue fluorescence (*λ* = 495 nm) of BCNMC was significantly enhanced after the encapsulation. In dark, the spiropyran moiety in BCNMC gradually isomerizes from a ring‐closed state to a ring‐open state. This isomerization facilitates intramolecular energy transfer from spiropyran to cyanostilbene, and thus alters the fluorescence from the initial blue to red (*λ* = 620 nm). The fluorescence can revert to its blue state under visible light irradiation due to reverse transformation of the piropyran moiety in BCNMC. The system can regulate fluorescence color via alternating light irradiation and dark conditions and also modify the topological morphology of the host‐guest complex between nanosheets and nanospheres. These unique optical properties enable applications in writable information encryption and multi‐color QR codes.

**FIGURE 2:**
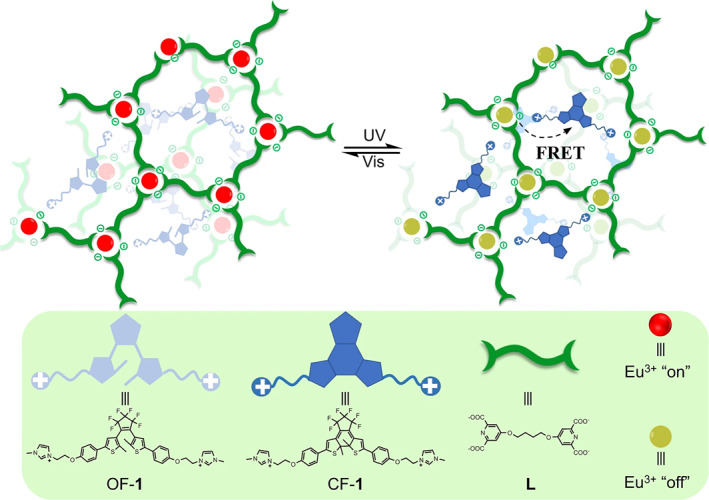
The construction of the photochromic supramolecular coordination polyelectrolyte, and the chemical structures of the corresponding components.

The PL color or intensity of a smart PL‐responsive system can also be regulated by altering the energy transfer pathway through photoisomerization of a particular photoswitch. Zhao and colleagues reported on light‐responsive supramolecular coordination polyelectrolytes, which are constructed via hierarchical self‐assembly of lanthanide ions, biligand units, and diarylethene units driven by metal‐ligand coordination and ionic interactions (Figure 2).^[51]^ These polyelectrolytes feature photoreversible light‐emitting switches are attributed to conformation‐dependent photochromic fluorescence resonance energy transfer (FRET) between lanthanide donors and diarylethene acceptors. This mechanism, which hinges on the closed‐loop/ring‐open isomerization of diarylethene units, paves the way for new intelligent anti‐counterfeiting materials.

**FIGURE 3:**
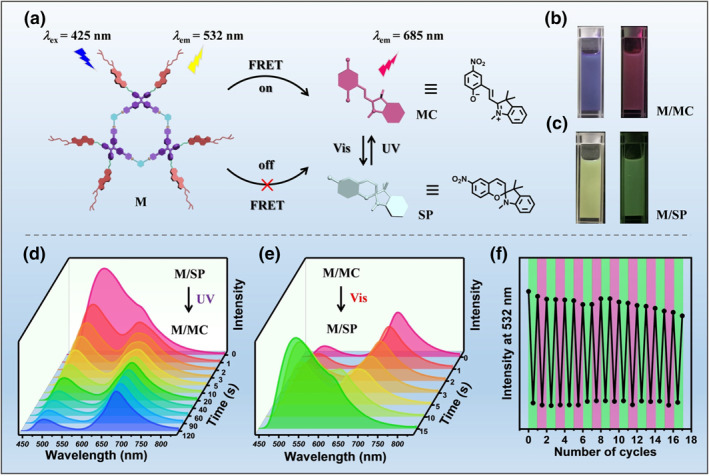
(a) Schematic illustration of the light‐responsive FRET system containing metallacycle **M** and spiropyran derivative **SP** or **MC**. (b) Photographs of **M/MC** in natural light (left) and 365 nm (right) in a nematic LC host. (c) Photographs of **M/SP** under natural light (left) and 365 nm (right) in the nematic LC host. (d) Fluorescence spectra of **M/SP** under UV irradiation over time (*λ*_ex_ = 425 nm). (e) Fluorescence spectra of **M/MC** under visible‐light irradiation for different times (*λ*_ex_ = 425 nm). (f) Changes in the fluorescence intensity of **M/SP** and **M/MC** at 532 nm upon alternating the UV/Vis irradiation cycles. Copyright 2023, Wiley‐VCH GmbH. LC, liquid crystal.

Cui et al. developed a novel photoresponsive dual‐light‐emitting material, ZJU‐128⊃SP (SP referring to spiropyran), by encapsulating SP within a cadmium‐based metal‐organic framework (MOF).^[52]^ This MOF/dye composite exhibits blue emission (*λ* = 447 nm) and red emission (*λ* = 650 nm) from the ZJU‐128 ligand and SP, respectively. Under Ultraviolet (UV) light irradiation, SP undergoes photoisomerization and thus opens a FRET channel from ZJU‐128 to spiropyran, resulting in a gradual decrease in blue emission and an increase in red emission. This reversible dynamic fluorescence behavior can be recovered entirely upon exposure to visible light (>405 nm). Utilizing this transient fluorescence, dynamic anti‐counterfeiting patterns and multiplexes based on the ZJU‐128⊃SP film can be achieved. Xu et al. developed a spatially controlled FRET system by incorporating a liquid crystal metal cycle with a photochromic spiropyran derivative.^[53]^ The hexagonal metal cycle M (Figure 3) decorated with tetraphenylene and cholesterol moieties was fabricated via coordination‐driven self‐assembly. The photochromic spiropyran derivatives are switchable acceptors. This system leverages light structural transitions between non‐emissive/colorless and emissive/blue‐conjugated forms of spiropyran derivatives, enabling photoswitchable changes in fluorescence color. Additionally, the light‐responsive FRET system allows orthogonal multimodal photopatterning of holography, fluorescence, and photochromism within a single integrated supramolecular system. This study represents a significant advancement in multimodal light patterning, promoting future developments in holographic, fluorescent, and photochromic photopatterning techniques.

**FIGURE 4:**
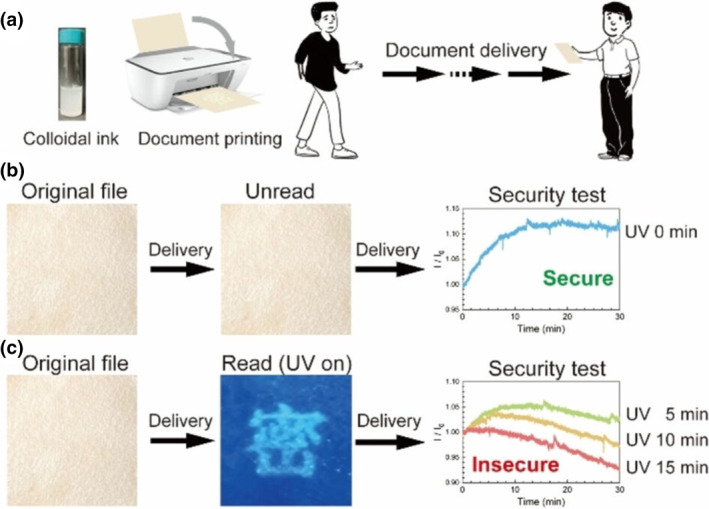
Polymer rods for encrypted printing, delivery, decryption and security check of files. (a) Schematic encrypted printing and delivery process. (b) Secure document delivery and security check. (c) Insecure document delivery and security check. In the rightmost of (b–c), they are fluorescence kinetic curves after they were irradiated by the UV light (365 nm) for 0, 5, 10 and 15 min. *Source*: Copyright 2023, Wiley‐VCH GmbH.

Another method of photo‐controlled fluorescence manipulation is the introduction of photolytic groups so that their fluorescence changes after UV illumination. Liu and colleagues successfully synthesized homogeneous colloidal polymer rods with tunable aspect ratios and well‐defined internal molecular packing and orientation through self‐assembly by stabilizer‐assisted liquids (Figure 4).^[54]^ These rods exhibited unusual photoinduced fluorescence enhancement and concomitant memory effects when irradiated with a UV lamp. This phenomenon originated from a complex formed between chain transfer agent (CTA) (4‐cyanovaleric acid dithiobenzoate) and triphenylene units during reversible addition‐fragmentation chain transfer polymerization. The triphenyl unit acts as a non‐emitting low‐energy exciton trap rendering the complex non‐fluorescent before exposure to light. However, when the complex is irradiated with UV light, the CTA breaks, thereby restoring the fluorescence of the quenched triphenyl unit. This property makes their dispersions applicable as advanced colloidal inks for encryption and the secure delivery of paper documents (Figure 4). Additionally, this mechanism holds great potential for other applications such as anti‐counterfeit labels.

### 3.2 Light‐controlled macroscopic shape/phase transformation

Drawing inspiration from biological tissues, many advanced synthetic soft materials have emerged, showing broad promise in regenerative medicine, targeted therapy, and soft robotics.[^55^, ^56^] These materials often swell or change shape in response to external stimuli.[^57^, ^58^] However, shape‐changing stimuli typically need to interact directly with the substrate, limiting the types of stimuli that can be used.[^59^, ^60^, ^61^] Light‐responsive supramolecules offer a more efficient strategy.[^62^, ^63^] B. L. Feringa and colleagues demonstrated a typical hydrogen‐bond‐based gel system in response to light irradiation, which can be reversible transformed between a solution state and a gel state.^[64]^ The key molecular design feature was the introduction of a very small number (2%) of a molecular photomotor into a supramolecular network as a photoswitched non‐covalent crosslinker. Bao et al. proposed a simple and universal method to obtain activated peptide hydrogels by introducing a photoremovable charge‐repulsive fragment, namely a 2‐nitrobenzyl photocleavable group.^[65]^ The core of this gelatinization strategy is to design a peptide gel with a high aggregation tendency and charge‐repulsive peptide.

**FIGURE 5:**
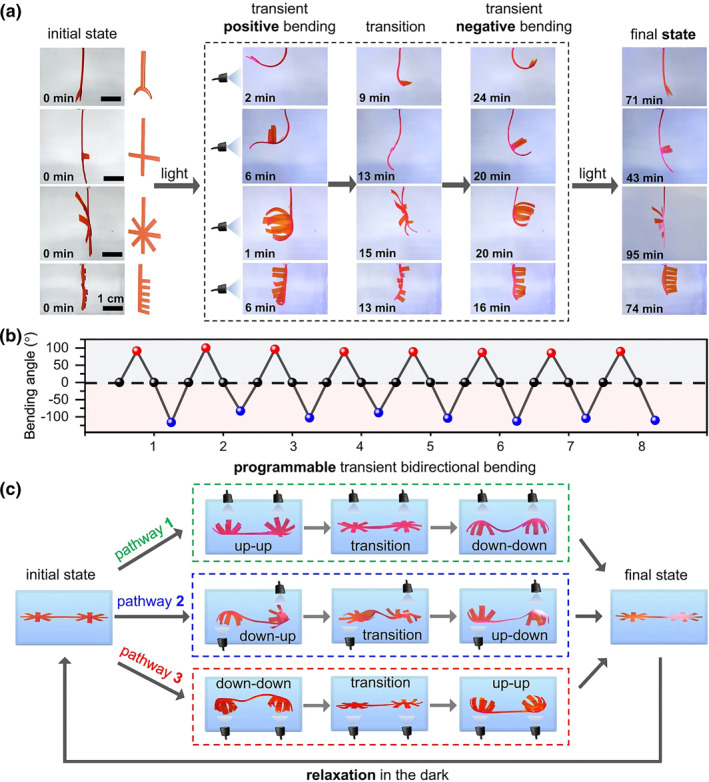
(a) Photographs of transient bidirectional bending deformation of hydrogels with multiple inverted shapes. (b) Plot of transient positive (red) and negative (blue) bending angles of a star‐shaped hydrogel for eight repeated cycles by alternatively switching light (450 nm, 15.24 mW/cm^2^) on and off. The hydrogel was incubated in acidic water for 8 h to fully relax to its original state. (c) Photographs of a single dual‐star‐shaped hydrogel that displays programmable transient deformation configurations by manipulation of different irradiation pathways.

The hydrophilicity of the polymer can be adjusted by changing their total charge which changes the water content in the hydrogel and thus leads to its deformation. Based on this principle, Li et al. reported a kind of hydrogel containing two kinds of spiropyrans, which exhibit self‐regulating deformation reversal when subjected to light irradiation (Figure 5).^[66]^ Two polymerizable spiropylan compounds were synthesized and designed to exhibit significantly different photoisomerization kinetics and completely opposite charge change abilities when irradiated with visible light. The change in total electrostatic charge affects the hydrophilicity of the polymer chains, driving water molecules in and out, which in turn leads to changes in the volume of the hydrogel. Hydrogel film actuators were developed to display complex temporary bidirectional shape transitions and self‐adjusting reverse scrolling under constant illumination. This work represents an innovative strategy to program complex shape transformations of homogeneous hydrogels using a single constant stimulus. Samuel I. Stupp's team reported a hybrid light soft material consisting of peptide amphiphilic supramolecular polymers.^[67]^ These polymers chemically bonded to helical pyran‐based networks and when photons enter the film the hydrophilic merocyanine (MCH^+^) moiety will be converted to a hydrophobic SP form, resulting in the expulsion of water, thereby shrinking the hybrid material. The bending of the materials is reversible because the MCH^+^ part is partially converted into SP when further illuminated. Supramolecular polymers form a reversible deformed and draining skeleton that mechanically enhances the hybrids. As a result, the non‐covalent skeleton embedded in the network enables faster bending and flattening of objects as well as longer step sizes in the light‐driven crawling motion of macroscopic films. This research shows that hybrid bonding polymers that integrate supramolecular covalent networks provide a strategy for the bottom‐up design of soft matter that mimics living organisms.

**FIGURE 6:**
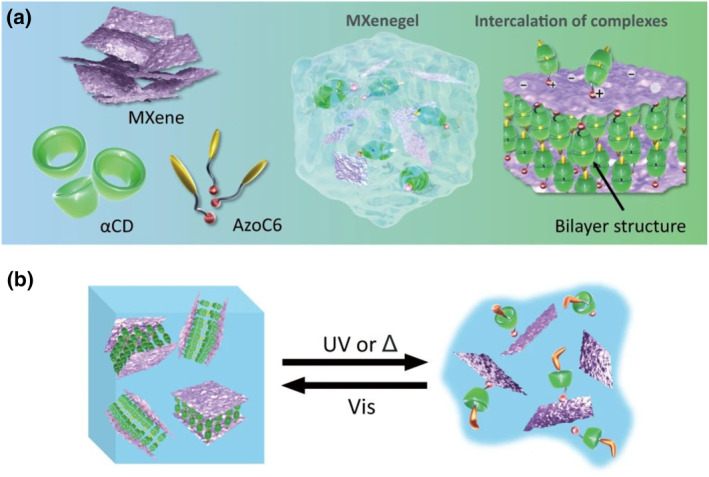
(a) The alpha‐cyclodextrin (α‐CD) host and the 1‐methyl‐3‐(5‐(4‐(phenyldiazenyl)phenoxy)pentyl)‐1H‐imidazol‐3‐ium (AzoC6) form a bilayer structure of AzoC6@2αCD complexes between the MXene nanosheets. Meanwhile, the positive head ends of the complexes were electrostatically assembled to the negatively charged MXene surfaces. (b) Light‐responsive sol‐gel transition behavior of the MXenegel.

Chen and co‐workers developed a light‐modulated MXenegel with reversible phase transitions based on light‐responsive host‐guest chemistry (Figure 6).^[68]^ MXenegel is prepared by weighing the required amounts of azobenzene derivatives (AzoC6) (**MS6**), α‐cyclodextrin (α‐CD), and MXene into an aqueous solution. MXenegel can undergo gel‐to‐sol transition under UV light irradiation or heat treatment, which is caused by the decomposition of AzoC6@α‐CD inclusions inserted between the MXene layers. The sol‐state MXenegel can be reformed back into a gelatinous structure under visible light or dark conditions at room temperature, suggesting that this phase transition is reversible.

**FIGURE 7:**
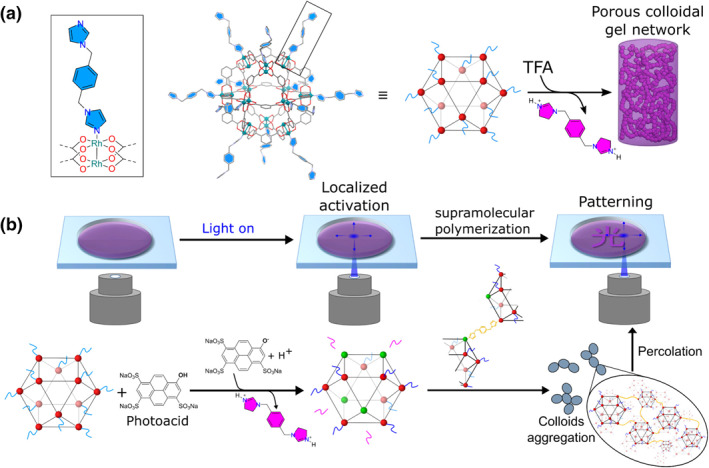
Strategy to achieve temporal and spatiotemporal control. (a) Schematic view of the molecular structure of the kinetically trapped MOP. (b) Schematic representation of the photopatterning experiment using confocal laser scanning microscopy and the corresponding supramolecular polymerization reaction when irradiating the solution of the aged MOP solution containing pyranine as photoacid. MOP, metal‐organic polyhedron. *Source*: Copyright 2021, American Chemical Society.

Photoacid is a light‐sensitive compound that can release protons under light irradiation. Employing photoacids in the materials, the pH‐sensitive physical and chemical properties of the materials can be regulated by light. Furukawa et al. demonstrated that the combination of light and photoacid allows spatiotemporal control of the structure, mechanical properties, and shape of metal‐organic polyhedron (MOP)‐based porous soft materials (Figure 7).^[69]^ With the addition of trifluoroacetic acid, a colloidal gel network can be formed from the MOP solution, and the concentration of the acid will affect the structure and mechanical properties of the gel. Pyranine **(MS7)** has been shown to locally change pH due to a decrease in pK_a_ from 7.3 to 1.4, and the self‐assembly process can be locally triggered by irradiation of the photoacid generator pyran which can control the formation of gels in time and space. Using this method, the gel can be patterned into the desired shape. The precise positioning of this assembled structure, with the stable and permanent porosity of MOPs, allows them to be integrated into devices for applications such as sensing, separation, catalysis, or drug release.

**FIGURE 8:**
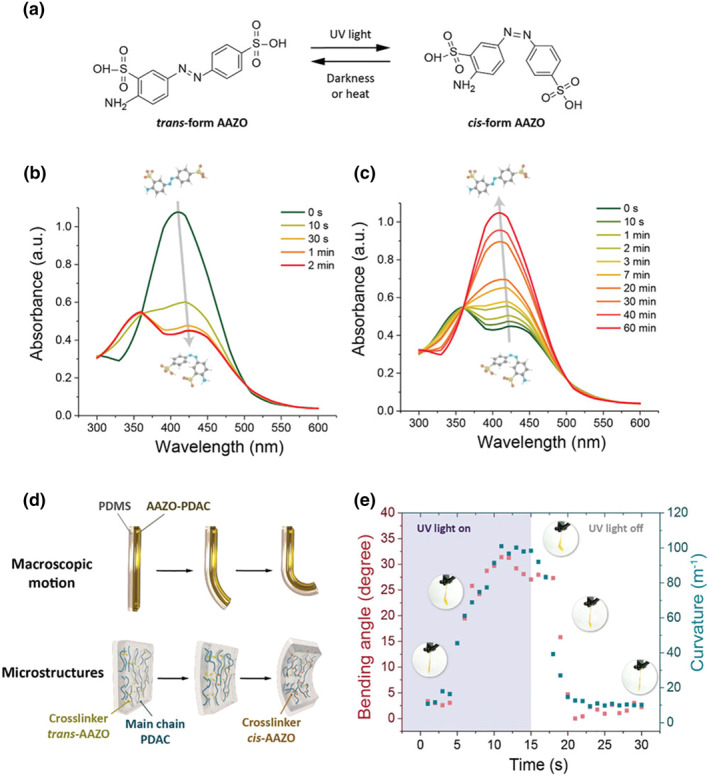
Photoswitching of the AAZO molecule and the photomechanical properties in the bulk state. (a) Reversible photoisomerization mechanism of the AAZO. UV–vis absorption spectra of AAZO solutions. (b) After shining UV light and (c) stored in darkness for different times. (d) Schematic illustration of the photo‐responsive deformation of a film controlled by different UV irradiation times in macroscopic and molecular scales. (e) Actual photographs and plot of bending angle and curvature versus time. *Source*: Copyright 2023, Wiley‐VCH GmbH.

Changing the conformation of polymers through photoisomerism can also drive the deformation of soft materials. Chen et al. proposed a universal design for fabricating protected cell‐inspired photoswitched ion channels by infiltrating azobenzene cross‐linked polymer (AAZO‐PDAC) into nanoporous anodized aluminum oxide (AAO) membranes(Figure 8).^[70]^ These azobenzene polymers are formed by the electrostatic interaction of azobenzene chromophore (AAZO) **(MS8)** cross‐linked poly(diallyl dimethylammonium chloride) (PDAC). Under UV irradiation, trans‐AAZO in the crosslinker isomerizes to cis‐ AAZO, resulting in volume compression and asymmetric internal stress of the AAZO‐PDAC layer, which ultimately leads to volume shrinkage of the polymer network. When placed in the dark, cis‐ AAZO reverts to trans‐AAZO, resulting in structural recovery. The infiltration of AAZO‐PDAC cross‐linked polymers into the nanochannels of the AAO template can control the pore size of the ionic nanochannels by photo‐driven, thereby controlling the ionic conductivity. These ion channels show great potential for applications in biomimetic materials, sensors, and biomedical science, providing a new approach to the creation of dynamic and responsive systems inspired by biological processes.

### 3.3 Light‐controlled microscopic morphologies regulation

In supramolecular chemistry, one of the significant challenges is linking phenomena at the molecular level to larger scales and ultimately to complex molecular systems that resemble living systems.[^71^, ^72^] Following the principle of molecular self‐assembly, numerous supramolecular microscopic topologies have been reported, such as one‐dimensional nanotubes, two‐dimensional nanosheets, vesicles, fibers, rings, and spirochetes.[^73^, ^74^, ^75^] These structural changes ultimately affect their function and performance, making photoresponsive supramolecular topology and topology manipulation highly promising for applications in devices and living systems.[^76^, ^77^] Hossein Alidaei‐Sharif and colleagues used spiropyran derivatives to modify the surface of functionalized latex nanoparticles (NPs).^[78]^ They employed semi‐continuous emulsifier‐free emulsion polymerization to prepare functionalized latex NPs with different particle sizes and morphologies, resulting in the development of invisible‐security anti‐counterfeiting inks. Jong‐Man Kim and colleagues efficiently captured metastable nanoribbons, nanocoils and nanohelices using chiral perylene imides with diethylene‐containing alkyl chains through UV‐facilitated polymerization.^[79]^ Luis Sánchez and colleagues report the self‐assembly of scissor‐shaped azobenzene binaries that form discrete nanorings and two‐dimensional porous networks. Photoisomerization of the azobenzene moiety allows for efficient dissociation/recombination of nanorings in solution.^[76]^

**FIGURE 9:**
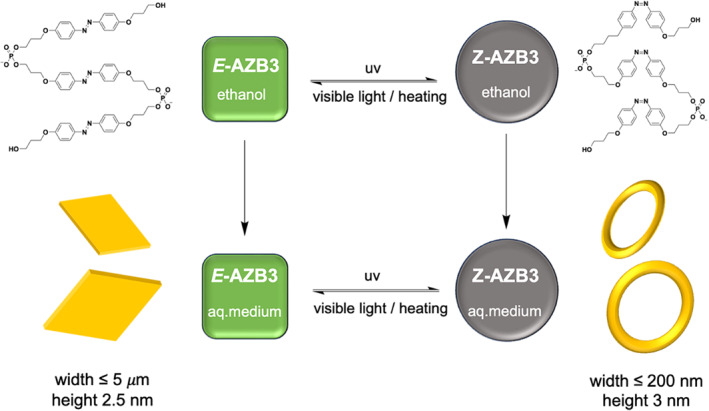
Reversible isomerization of ***E‐AZB3*** to ***Z‐AZB3*** trimer upon UV‐light irradiation and vice versa upon irradiation with visible light or heating, and light‐triggered reversible interconversion of SPs with different morphologies. UV, Under ultraviolet. *Source*: Copyright 2021, The Authors. Angewandte Chemie International Edition published by Wiley‐VCH GmbH.

In addition, the combination of photoisomeric molecules with amphiphilic molecules is a powerful way to regulate the microstructure of supramolecules. Robert Häner et al. prepared an amphiphilic, phosphodiester‐linked azobenzene trimer (AZB3) **(MS9)** that can undergo reversible E‐Z isomerization both thermally and photochemically(Figure 9).^[80]^ The negative charge of the phosphate group ensures water solubility, while the hydrophobicity of the AZB unit promotes folding and assembly of trimer in an aqueous environment. In aqueous media, the E‐AZB3 trimer self‐assembles into micrometer‐sized two‐dimensional nanosheets, while the Z‐AZB3 trimer forms a circular nanostructure. Photoisomerization of AZB by exposure to UV or visible light allows for reversible destruction or recovery of two‐dimensional nanosheets, paving the way for new stimuli‐responsive water‐soluble supramolecular hydrogels, crucial for developing smart functional materials. Similarly, Bumjoon J. Kim et al. reported block copolymer (BCP) particles that exhibit reversible shape and color changes when activated by ultraviolet (UV) and visible light irradiation.^[81]^ The SP group can isomerize between its hydrophobic ring‐closed SP form (RC form) under visible light and the hydrophilic ring‐opened merocyanine form (RO form) under UV irradiation. This wavelength‐dependent isomerization of the SP‐DTAB surfactant enables reversible changes in its interfacial activity between the polystyrene (PS) and poly(4‐vinylpyridine) (P4VP) blocks in the BCP particles. It allows the shape transformation of BCP particles between spherical onion‐like particles with a P4VP outermost layer and ellipsoidal particles with both PS and P4VP exposed to the surface of the particles. Notably, this change in BCP particle morphology between spheroids and spheroids is reversible over multiple cycles of UV and visible light irradiation. Furthermore, a free‐standing hydrogel film was fabricated by incorporating the oblate PS_27_k‐b‐P4VP_7_k particles into a hydrogel matrix, and the reversible patterning capability of the hydrogel film was demonstrated. This innovative approach allows for dynamic and tunable materials that can respond to light stimuli, making them suitable for applications in advanced display technologies and various other fields requiring responsive materials.

**FIGURE 10:**
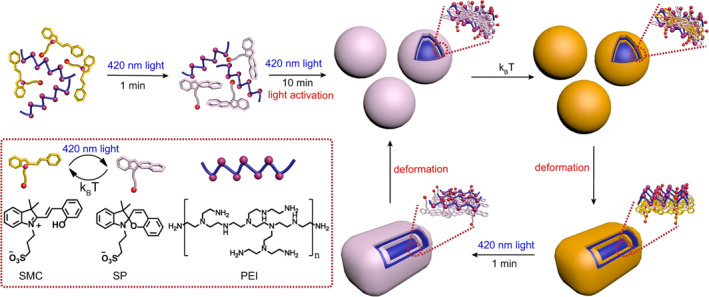
Light‐activation process for dissipative self‐assembly (initial two steps), and reversibly photodeformable dissipative self‐assembly under light irradiation/thermal relaxation cycles (following steps in the loop). The schematic and chemical structures of SMC, SP, and PEI are shown in the dashed box. PEI, polyethylenimine; SMC, sulfonic acid‐merocyanine.

Influencing electrostatic interactions by changing molecular charge is another way to regulate the morphology of supramolecular assembly. Li et al. reported a light‐activated photodeformable dissipative self‐assembly system in aqueous solution as metastable fluorescent palette (Figure 10).^[82]^ At 420 nm light irradiation, zwitterionic sulfonic acid‐merocyanine (SMC) isomerizes to spiropyranoic acid, subsequently ionizing into negatively charged sulfonic‐spiropyran (SP) (**MS10**) and H^+^. The resulting H^+^ is captured by the amino group from polyethylenimine (PEI) forming positively charged PEI. This leads to strong electrostatic interaction and hydrophobic effects that form transient spherical supramolecular NPs. After the light irradiation is removed, SP begins to revert to SMC. The high positive charge density of PEI maintains a strong electrostatic interaction with SP, resulting in a ternary supramolecular co‐assembly system of SMC‐SP‐PEI, forming larger cuboid NPs. These systems can be reversibly converted between SP‐PEI spherical NPs and SMC‐SP‐PEI cuboid NPs through light irradiation and thermal relaxation, showcasing the potential for more advanced applications.

**FIGURE 11:**
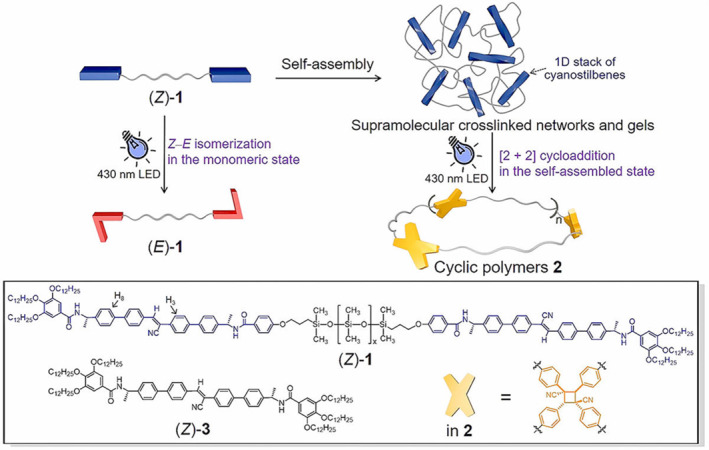
Supramolecular confinement‐induced cyclic polymerization of (*Z*)‐**1**upon light irradiation. The chemical structures of (*Z*)‐**1**, (*Z*)‐**3**, and the [2 + 2] photo‐cycloaddition units in cyclic polymers **2** are shown in the frame. *Source*: Copyright 2021, Wiley‐VCH GmbH.

Another strategy is to produce a certain confinement effect on the compound through supramolecular packing, so as to obtain the desired target compound structure. Wang et al. designed a remote chelated large monomer (Z)‐1 (**MS11**) with terminal functionalized cyanostilbene (Figure 11),^[83]^ in which the amide groups at the end of the cyanostilbene unit form highly directed hydrogen bonds, resulting in supramolecular crosslinking networks and gels in nonpolar solvents. Unlike the Z‐E isomerization of cyanostiblene in the monomeric state, the supramolecular assembly of (Z)‐1 exhibits a limiting effect, so [2 + 2] halo addition is preferred under 430 nm light irradiation to form cyclic polymers. This approach highlights the potential for precise control of photochemical selectivity through supramolecular confinement, opening up new avenues for efficient cyclic polymer synthesis.

### 3.4 Light‐controlled chiral regulation

**FIGURE 12:**
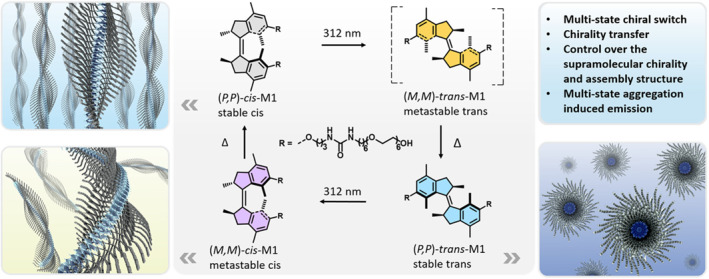
Molecular structures of molecular motor **M1** and illustration of multistate rotation, isomers with distinct chirality, and the corresponding assembly structures.

Chirality is one of the most essential and fundamental characteristics of nature, and controlling hierarchical chirality is key to complex biological processes in organisms, which rely on a delicate balance between molecular, supramolecular, and macroscopic chirality.[^84^, ^85^, ^86^, ^87^, ^88^, ^89^, ^90^, ^91^, ^92^] Changing the conformation through the isomerization of small molecules and thus altering their chirality is one common method for chiral control. B. L. Feringa's team proposed a light‐driven supramolecular polymer molecular motors (**MS12**), in which intrinsic chirality is transferred to nanofibers. The rotation of the molecular motor controls the chirality and morphology of the supramolecular polymer, realizing dynamic control of the supramolecular polymer in multiple self‐assembly states (Figure 12).^[93]^ Starting with spiral, the morphology converts into micelles, followed by worm‐like micelles, and finally restores the spiral fibers. The resulting supramolecular polymers also exhibit light‐controlled polymorphic aggregation‐induced emission. The emission of the supramolecular polymer was assumed to be attributed to the restriction of the excited‐state rotation of the molecular motor in confined space.

**FIGURE 13:**
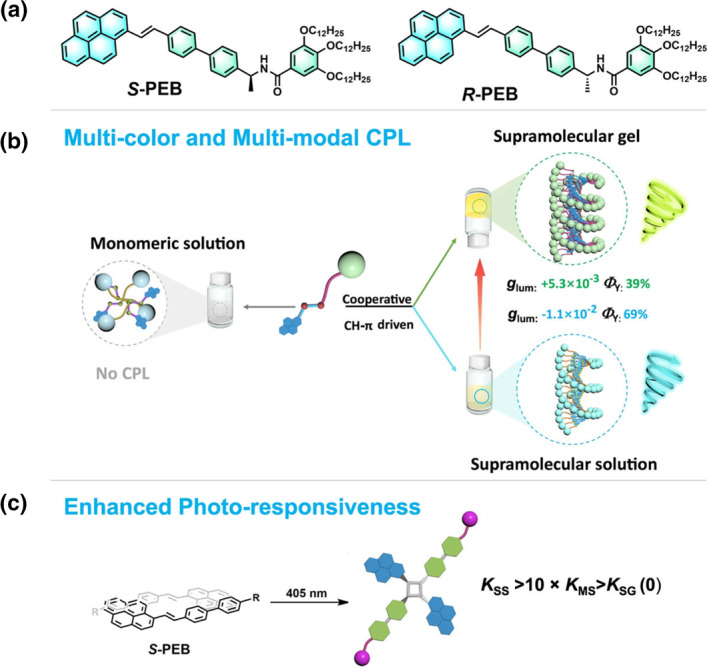
(a) Molecular structures of *S‐*PEB and *R‐*PEB. (b) Multi‐color and multi‐modal CPL properties of the *S‐*PEB in various assembled states. (c) Different [2 + 2] cycloaddition reaction rates *K*. *K*_SS_ for the supramolecular solution, *K*_MS_ for the monomeric solution, and *K*_SG_ for the supramolecular gel. CPL, circularly polarized luminescence.

Yuan et al. explored another intriguing method for modulating chirality through the degree of dissociation after photocyclization, constructing a one‐component circularly polarized luminescence (CPL) material based on styrylpyrene (Figure 13).^[94]^ Utilizing CH‐π interactions, styrene‐based pyrene core molecules form long‐range ordered helical fibers. This leads to the creation of supramolecular gels and thin film samples with excellent CPL performance. One of the remarkable features of this material is its ability to change the emission color and chirality depending on its state. From supramolecular solutions to gels or films, the emission color changes from blue to yellow or green. Concurrently, the chirality of the CPL is reversed although the supramolecular chirality remains unchanged. This unique makes it a multimodal and color‐dependent one‐component CPL active material. Additionally, the supramolecular confinement effect significantly influences the reaction rates of light‐induced [2 + 2] cycloaddition reactions. The reaction rate in the supramolecular solution is 10.5 times that of the monomer solution. In contrast, gels and self‐assembled solids do not undergo cycloaddition reactions. This selective reactivity showcases the potential for designing versatile CPL active materials with controllable CPL color and chirality. The ability to modulate chirality and emission through supramolecular interactions and state changes opens up new avenues for the development of chiral light‐responsive devices. These materials can be employed in various applications, including advanced optical materials, sensors, and display technologies.

**FIGURE 14:**
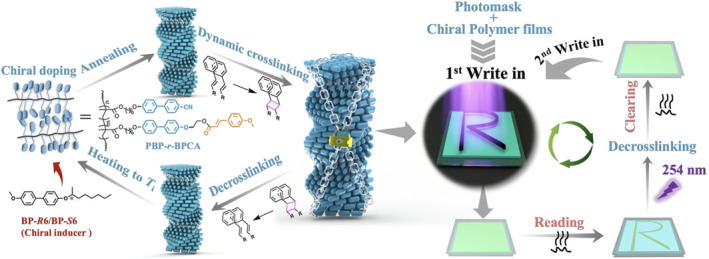
Illustration of the dynamic fixation process and pattern‐embedded storage mechanism in chiral polymeric superstructures. *Source*: Copyright 2023, Wiley‐VCH GmbH.

Constructing chiral superstructures through the self‐assembly of achiral polymers usually relies on the interaction of multiple non‐covalent bonds. Introducing covalent crosslinking can undoubtedly enhance the stability of chiral superstructures, Zhang et al. reversibly immobilized supramolecular chirality by introducing dynamic covalent cross‐linking into the chiral self‐assembly of side‐chain polymers (Figure 14).^[95]^ Non‐chiral side‐chain liquid crystalline polymers containing biphenyl groups were introduced to be a chiral induction model and cinnamate structure to be a dynamic crosslinking group in side chains. The reversible [2 + 2] cycloaddition reaction of cinnamate in the polymer chain can be further controlled by light, manipulating interchain cross‐linking and de‐cross‐linking. Based on this optically programmable and dynamic chiral fixation strategy, a new mode‐embedded storage mechanism for chiral polymer materials was established. This innovative approach of chiral transfer, modulation, and memory paves the way for the precise construction and application of chiral polymer materials in the future.

**FIGURE 15:**
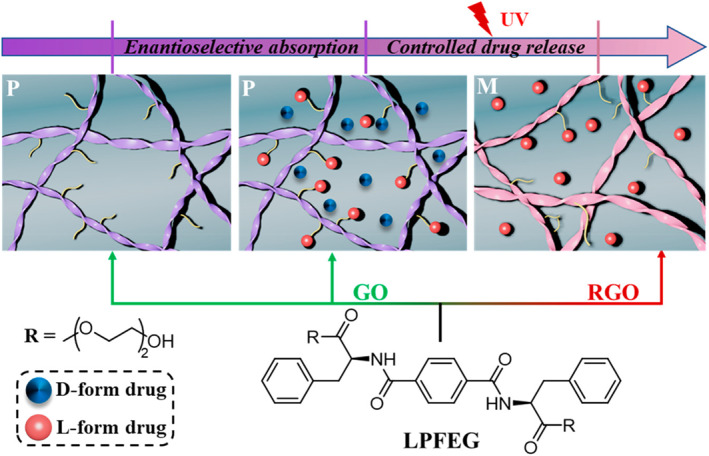
Chirality and morphology of co‐assembled nanostructures and the chirality inversion as well as the corresponding enantioselective delivery of chiral drugs are triggered by UV irradiation. *Source*: Copyright 2020, American Chemical Society.

Feng et al. provide an example of controlling the chiral of supramolecular hydrogel, where the combination with graphene oxide (GO) results in the opposite helix of the L‐phenylalanine derivative gel (LPFEG) (**MS15**) by controlling light exposure and temperature (Figure 15).^[96]^ LPFEGs are assembled in different ways in combination with GO and reduced GO (RGO). Only right‐handed helical fibers were initially formed binding to GO, while preferred left‐handed nanofibers were observed when GO was reduced by UV irradiation or heating. The combinatorial gel has GO‐related stimulus‐responsive properties enabling irradiated or heat‐driven spiral chiral inversion. This supramolecular system provides insights into surface chiral assembly and offers an efficient and simple way to achieve precisely controlled enantioselective drug‐free absorption and release.

### 3.5 Light‐controlled targeted drug delivery

Light‐responsive supramolecular systems can be applied to controlled local drug delivery, opening up new horizons for tissue engineering and regenerative medicine.[^97^, ^98^, ^99^, ^100^, ^101^, ^102^, ^103^] You et al. demonstrated a versatile optical switch covalent cascade platform for remote and bidirectional control of reversible covalent bonds subsequent assembly simulating biological signal cascades.^[104]^ Tian et al. developed photochromic fluorescent sugar stereotypes capable of remotely controlling intracellular target identification.^[105]^ Amphiphilic glycoprobes that form micelles in water can selectively enter target cells and undergo photochromic cycles under the control of alternating UV/Vis irradiation. Fang et al. developed a metallic phenolic network‐integrated multi‐level nanosystem for enhanced NP penetration and drug delivery in tumors, achieved through light‐triggered nanostructure disassembly.^[106]^

**FIGURE 16:**
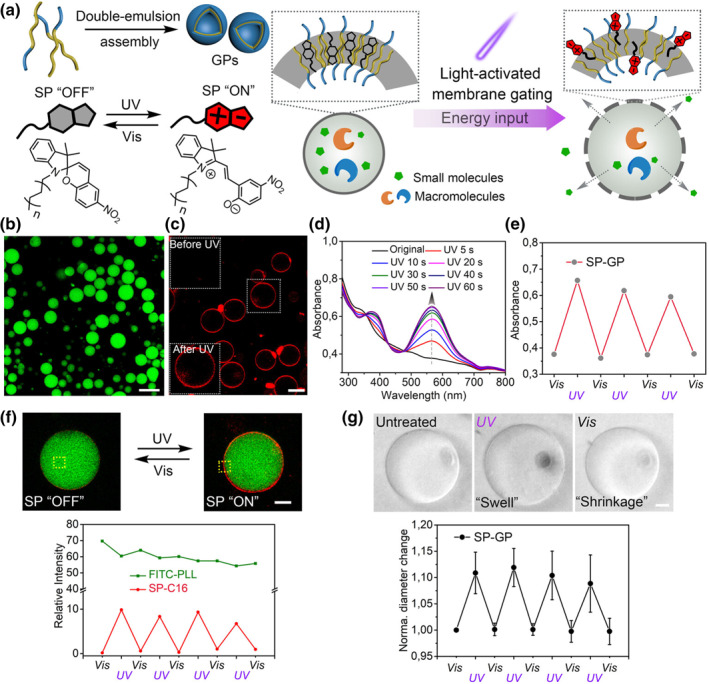
(a) Upon UV‐light irradiation, the fast isomerization of the SP resulting in reversible swelling and increased membrane permeability. (b) The GPs can compartmentalize a hydrophilic cargo in the aqueous core. (c) The SP initially did not show fluorescence emission and turns on emission upon UV irradiation. (d) UV/Vis absorbance of spiropyran‐embedded polymersomes before and after UV irradiation as a function of exposure time. (e) Reversible change in UV/Vis absorbance intensity of SP‐GPs at λ_abs_ = 560 nm produced by alternating UV/Vis irradiation. (f) Reversible fluorescence of SP‐GPs membrane upon UV/Vis irradiation. (g) The photo‐isomerization of SP‐GPs induced a reversible mechanical response. UV, Under ultraviolet. *Source*: Copyright 2022, The Authors. Angewandte Chemie International Edition published by Wiley‐VCH GmbH.

Another approach to controlled drug is altering the interfacial properties of NPs enabling on‐demand drug release. Landfester et al. developed a supramolecular giant polymer (GP) with photoactivatable membrane permeability, useful for constructing light‐controlled cell membrane mimics, engineered synthetic spiropyran‐based permeability modulators **(MS16)** for integration into impervious GP membranes (Figure 16).^[107]^ It was found that the photoisomerization of spiropyran (SP) could significantly improve the membrane permeability of SP‐GPs. This effect allows small hydrophilic molecules to pass through the polymer membrane of SP‐GPs only when the vesicles are exposed to UV. This photoinduced control of membrane permeability opens up potential applications in targeted drug delivery and controlled release systems where the release of encapsulated substances can be precisely regulated by light exposure. They also built a light‐activated enzyme microreactor where the substrate present in the external medium crosses the membrane barrier only after permeabilizing the GP by light irradiation, initiating an enzyme‐mediated reaction within the compartment. The design principles of this work will facilitate the construction of bottom‐up artificial structures with increasing biological complexity for biotechnological applications. Shi et al. reported a straightforward universal method to generate light‐responsive microcapsules by assembling supramolecules at the oil‐water interface.^[100]^ Switching the NP surfactant from the jammed state to the unjammed state by light triggering effectively controls the permeability of the colloidal membrane, thereby controlling the diffusion and release of molecules. This provides a multifunctional platform for building next‐generation intelligent interface systems.

**FIGURE 17:**
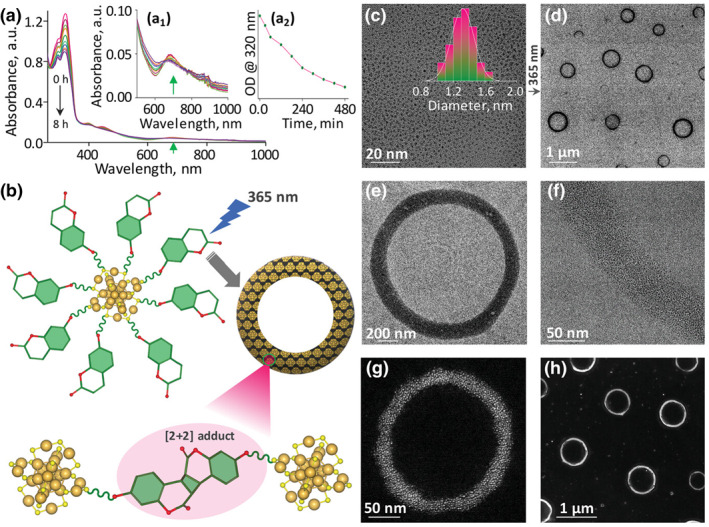
(a) Temporal UV–vis absorption spectra of NCs recorded under 365 nm illumination. Insets a_1_ and a_2_ show an enlarged view of the HOMO‐LUMO band as well as changes in OD as a function of illumination time. (b) A schematic showing the photodimerization and self‐assembly of Ncs. (c,d) TEM micrographs of NCs (c) before, and (d) after 365 nm lighting. (e,f) Zoomed‐in and focused HRTEM micrographs of (e) a single toroid, and (f) the edge of a toroid. (g,h) STEM images of (g) a magnified view of a single toroid, and (h) large area toroidal self‐assembly. Ncs, nanoclusters; UV, Under ultraviolet. *Source*: Copyright 2023, Wiley‐VCH GmbH.

Another effective strategy for light‐controlled drug release is regulating the assembly of nanoclusters. Shibu et al. demonstrated reversible [2 + 2] photocyclization reaction‐assisted self‐assembly of coumarin‐protected atomically precise Au25 nanoclusters (NCs) (atomic‐level precision NCs) (**MS17**) (Figure 17).^[108]^ Irradiation of the NCs at 365 nm facilitates inter‐NC coupling by cycloaddition reaction, resulting in a homogeneous annular superstructure. Transient spherical structures formed undergo fusion and transform into rings. Constant light exposure results in inter‐annular coupling at the supercolloid level forming a macroscopic honeycomb structure. For light‐controlled drug delivery, they demonstrated an intermolecular halo addition reaction between coumarin‐tethered NC and 5‐fluorouracil (an anticancer drug), forming a light‐controlled drug delivery system. When exposed to 254 nm light, the superstructure loaded the drug fragments into individual NCs and drug molecules, demonstrating photoreversibility. These NCs with improved photostability and photosensitizing functional ligands are expected to provide new avenues for combined therapeutics, diagnostics, and advanced drug delivery.

**FIGURE 18:**
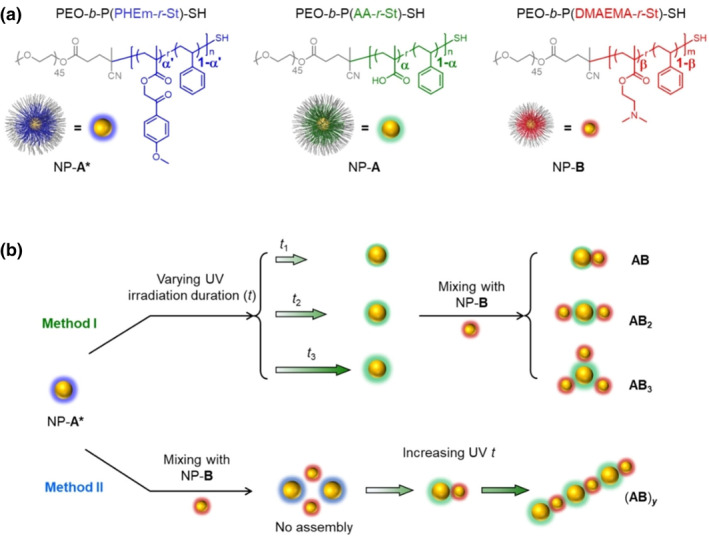
Schematic illustration of the photo‐induced self‐assembly of copolymer‐capped Au NPs to form colloidal molecules. (a) Under 254 nm UV irradiation, NP‐A*s with *p*‐methoxyphenacyl ester groups are transformed into NP‐**A**s carrying carboxylic acid groups which can react with dimethylaminoethyl groups of NP‐**B**s. (b) Schematic illustration of the two distinct self‐assembly pathways. Method I: NP‐A*s are irradiated to generate NP‐**A**s before being mixed with NP‐**B**s. *T*_*1*_∼*t*_*3*_ is varying the irradiation duration. Method II: NP‐A*s and NP‐**B**s were mixed before UV irradiation. UV, Under ultraviolet. *Source*: Copyright 2023, Wiley‐VCH GmbH.

Sang and Nie et al. developed a photocracking strategy for copolymer ligands to achieve targeted drug delivery (Figure 18).^[109]^ Under UV irradiation, the homogeneous C−O bond of benzoyl ester compounds was broken to form acryloxy radicals and benzyl derivative radicals. The formed p‐methoxyphenacyl is chemically unstable and undergoes rapid transfer of H atoms in the presence of a hydrogen bond donor to produce a carboxyl group, reacting with the tertiary amine of the copolymer on NP‐B to trigger a directed NP. By varying the duration of irradiation and thus the number of carboxyl groups produced on NP‐A (**MS18**), they can precisely control the x‐value of AB_x_ between 1 and 3. When NP‐A* and NP‐B are mixed and irradiated, the assembly process produces clusters or linear (AB)_y_ structures with sequential alternation of binary NPs. This assembly method provides a simple and non‐invasive way to externally modulate the formation of colloidal molecules as needed, without redesigning the surface chemistry of NPs for applications in drug delivery, diagnostics, optoelectronics, and plasmonic devices.

### 3.6 Light‐controlled microrobots

**FIGURE 19:**
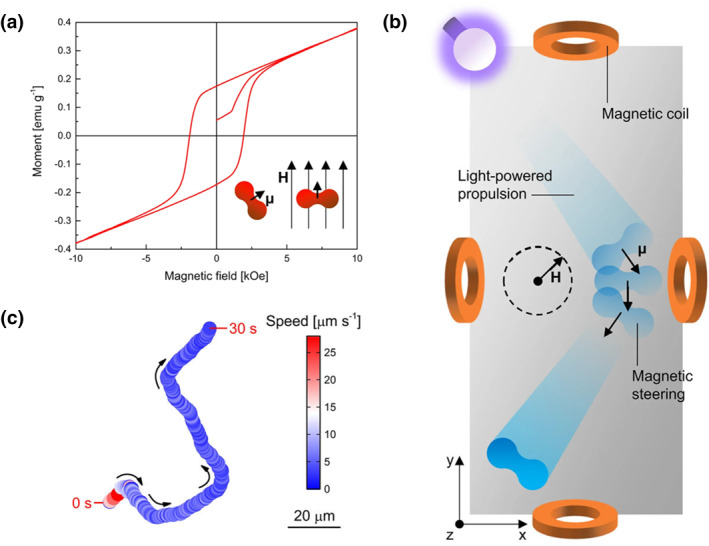
(a) Magnetic hysteresis loop of α‐Fe_2_O_3_ microparticles. The inset illustrates the magnetic dipole moment (μ) of an α‐Fe_2_O_3_ microparticle and its orientation according to the direction of an applied magnetic field (H). (b) Scheme of the magnetic steering of a TiO_2_/α‐Fe_2_O_3_ microrobot under UV light irradiation using the rotation of a magnetic field (H) while their direction is continuously adjusted. (c) Representative trajectory of a TiO_2_/α‐Fe_2_O_3_ microrobot with decreasing instantaneous speed (color‐coded) under simultaneous application of UV light irradiation and a magnetic field. UV, Under ultraviolet.

Light‐driven micro/nanobots are miniaturized, untethered machines with great potential in precision medicine, nanofabrication, and various other fields.[^110^, ^111^] Their usefulness hinges on developing light manipulation strategies that offer versatility, flexible design options, and precise controllability.[^112^, ^113^] Light‐driven microrobots can exhibit propulsion and different motion modes, which present an exciting area of exploration.[^114^, ^115^, ^116^] The photoinduced electron transfer process enables the generation of a concentration gradient of charged products, which is a powerful means for constructing light‐driven nanorobots. Based on this principle, Martin Pumera's group demonstrated the interaction control, reconfigurability, reversibility, and active self‐assembly of the TiO_2_/α‐Fe_2_O_3_ microrobot (Figure 19).^[117]^ The microrobot consists of peanut‐shaped α‐Fe_2_O_3_ microparticles synthesized using a hydrothermal method and covered with a thin layer of TiO_2_ via atomic layer deposition. Under UV light, the TiO_2_ layer absorbs photons with sufficient energy to excite electrons from the valence band of the semiconductor. The electron‐hole pairs react with surrounding water to produce a charged product concentration gradient, fueling the autonomous movement of the microrobot through electrolyte self‐diffusion electrophoresis. Removal of the photostimulation results in cluster dispersion and reconfiguration into a serpentine microchain of adjacent microrobots held together by magnetic dipole‐dipole interactions. These microrobots can switch from one self‐organizing state to another multiple times with UV light activation or deactivation. This research lays the groundwork for developing more complex meta‐that mimic nature. Moreover, this group developed the spontaneous assembly of hematite‐based microrobots with different shapes.^[118]^ Autonomous motion‐light‐driven hematite/Pt microrobots with cubic and walnut‐like shapes were fabricated by hydrothermal synthesis. Upon UV light exposure, charge occurs within hematite, transferring electrons from the hematite conduction band to the Pt layer. Protons are produced by water oxidation on the hematite side and consumed on the Pt side, establishing a proton gradient that creates an electroosmotic flow, causing a net displacement of particles propelling the microrobots with hematite as the front side. These microrobots exhibit different synchronous movements under light irradiation, enabling them to perform multiple tasks such as capturing, picking, and transporting microscale objects, degrading polymer materials, and remediating wastewater.

**FIGURE 20:**
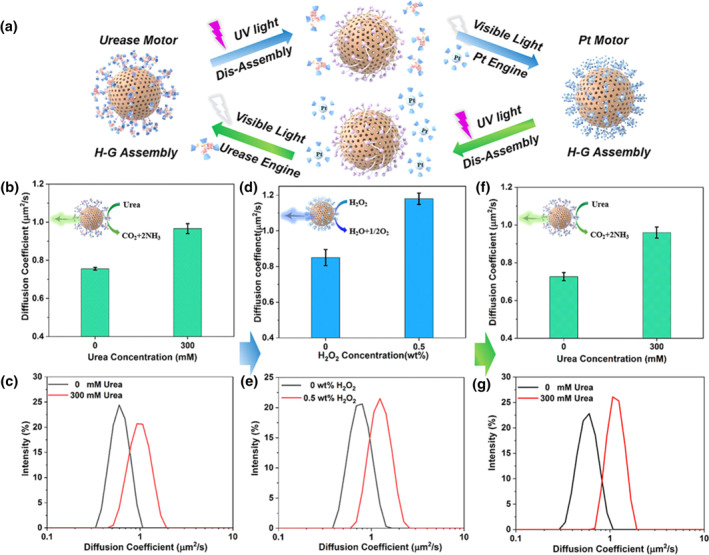
Cyclic switching of the nanoengine by light‐responsive disassembly and reassembly of host–guest interactions. (a) Schematic illustration of the disassembly and reversible assembly of nanoengines to achieve switching between a urease motor and a Pt motor through UV and visible light irradiation in turn. Diffusion coefficient and distribution (by dynamic light scattering) of (b,c) the initial urease motor, (d,e) the Pt motor after switching Pt engines, and (f,g) the urease motor after reassembling the urease engine again. Error bars indicate standard deviation (*N* = 10). *Source*: Copyright 2021, American Chemical Society.

Constructing nanomotors with replaceable engines based on host‐guest assembly/disassembly of supramolecular machines is another effective strategy to expand the versatility of microrobot applications. Ma and co‐workers demonstrated control of engine replacement of self‐propelled nanomotor based on hollow mesoporous silica nanoparticles (HMSNP) through supramolecular machine‐based master‐guest assembly and disassembly between azobenzene (Azo) (**MS19**) and β‐cyclodextrin (β‐CD) (Figure 20).^[119]^ By corresponding β‐CD‐modified nanoengines (urease, platinum, or Fe_3_O_4_) and assembling them with azobenzene‐modified HMSNPs (HMSNPs‐Azo), nanomotors with different driving mechanisms were rapidly constructed. The light‐controlled cis‐trans isomer conversion of the azobenzene molecule enables remote light‐triggered master‐guest assembly or disassembly between HMSNP azo and β‐CD‐modified engines, facilitating engine switching. This strategy is cost‐effective, allows rapid and convenient nanomotor fabrication with varied propulsion mechanisms, and paves a new path for the multifunctionality of microrobots, enabling on‐demand tasks in the future.

**FIGURE 21:**
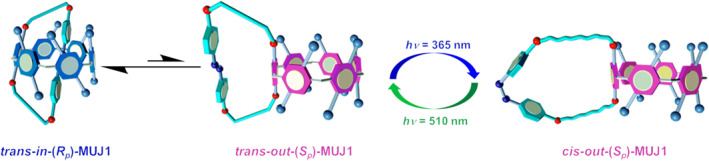
Suppositional mechanism of light‐driven chirality switching of (*in‐R*_p_/*out‐S*_p_)‐**MUJ1**.

Another approach involves changing the conformation of molecules through photoisomerism, which in turn controls the entry and exit of other molecules. Yang reported the construction of a smart molecular chiral optical switch based on azobenzene‐fused double‐ringed[n] aromatic derivatives, which defined as a molecular universal joint (**MS20**) (Figure 21).^[120]^ Cis‐azobenzene is bulky and therefore cannot be accommodated in the Pillar[6]arene cavity, so it rolls out of the cavity when azobenzene suffers photoisomerization from trans to cis, resulting in planar chiral switching of MUJs. Additionally, temperature changes can lead to conformational/chiral inversion due to significant entropy changes during ring flipping. When the temperature exceeds the upper limit, light‐induced chiral switching is disabled, thereby providing an over‐temperature protection function. This differs from the common low‐temperature gating effect. This study achieves a challenging high‐temperature gating effect at the molecular level and represents a significant step in the construction of intelligent microrobots capable of performing complex functions. Similarly, Qu et al. successfully combined a photochemically driven motor with the host‐guest chemistry of the crown ether.^[121]^ A series of electrokinetic macrocycles were synthesized by intramolecular cyclization of the first‐generation molecular motor with oligoethylene glycol chains. The photochemically driven rotation of the embedded molecular motor can be realized at the same time without affecting its unidirectional rotational motion and the formation of multiple states as well as effective host‐guest binding. The photoisomerization process can reversibly adjust the conformation of the crown ether moiety, resulting in a significant photoswitch binding affinity with the dialkylammonium guest molecule. The results show that there are significant differences between cis and trans isomers in the host‐guest recognition of the motorized macroring, suggesting that the guest can be captured or released in situ by changing the geometry of the motorized macroring. The combination of molecular motors, macrocyclic host, and guest chemistries serves as an excellent starting point for designing and building motor macrocyclic and more complex artificial molecular switches and machines. These advancements highlight innovative ways to develop light‐responsive systems at the molecular level, contributing to the creation of more sophisticated and versatile microrobots and molecular machines capable of dynamic task performance and complex functionalities.

## 4 SUMMARY AND OUTLOOK

In summary, research on LCSMs has progressed rapidly. By integrating photo‐sensitive molecular motifs with various supramolecular functional materials, scientists have developed numerous LCSMs with multiple responses. These materials not only exhibit unique photoresponsiveness but also leverage the advantages of supramolecular systems, such as dynamics, reversibility, and ease of construction. The reported LCSMs have shown potential applications in information storage encryption, micro/macroshape and phase transformation, medical drug delivery, robotics, and so on. However, despite these advancements, several challenges limit the development of this field. Firstly, the types of photoresponsive molecular motifs available for constructing LCSMs are limited. The core photoresponsive molecules used in LCSMs are photoswitch molecules such as azobenzene and spiropyran. While these molecules possess excellent photoreversible responses and are ideal for designing LCSMs, the lack of novel photoswitch molecules has become a significant constraint. Thus, designing and developing new molecules with superior photoreversible responses at the molecular level remains a challenge for photochemists and photofunctional materials scientists. Secondly, the mechanisms of photoresponsive molecules primarily involve the photoinduced reconstruction, formation, and cleavage of covalent bonds. In this regard, exploring non‐covalent interactions within supramolecular assemblies and enabling light to act directly on these interactions, causing them to generate or be destroyed, will significantly enhance the scope of light‐controlled supramolecular systems. Thirdly, the response level of existing LCSMs to light is relatively low. Most systems can only respond to light once or reversibly. From a practical and biomimetic perspective, developing smart material systems that can respond to various light sources or exhibit a multi‐level step‐by‐step response to light will make LCSMs more intelligent. In short, the advancement of LCSM hinges on the development of new photoresponsive molecules and supramolecular systems. Creating systems with richer stimulus responses and more diverse response mechanisms at the supramolecular level is essential for supporting the further development of the LCSMs.

## CONFLICT OF INTEREST STATEMENT

The authors declare no conflicts of interest.

## Data Availability

Data sharing not applicable—no new data generated or the article describes entirely theoretical research.

## References

[1] M. Su , Y. Song , Chem. Rev. 2022, 122, 5144. 34415152 10.1021/acs.chemrev.1c00303

[2] L. Tang , L. Wang , X. Yang , Y. Feng , Y. Li , W. Feng , Prog. Mater. Sci. 2021, 115, 100702.

[3] Y.‐J. Kim , Y. T. Matsunaga , J. Mater. Chem. B 2017, 5, 4307. 32263961 10.1039/c7tb00157f

[4] T. Bian , Z. Chu , R. Klajn , Adv. Mater. 2020, 32, 1905866. 10.1002/adma.201905866 31709655

[5] Z. Li , H. Chen , B. Li , Y. Xie , X. Gong , X. Liu , H. Li , Y. Zhao , Adv. Sci. 2019, 6, 1901529. 10.1002/advs.201901529 PMC6839628 31728289

[6] M. Regehly , Y. Garmshausen , M. Reuter , N. F. König , E. Israel , D. P. Kelly , C.‐Y. Chou , K. Koch , B. Asfari , S. Hecht , Nature 2020, 588, 620. 33361791 10.1038/s41586-020-3029-7

[7] S. Kobatake , S. Takami , H. Muto , T. Ishikawa , M. Irie , Nature 2007, 446, 778. 17429396 10.1038/nature05669

[8] D. Huang , J. Yin , Y. Zou , H. Huang , S. Long , W. Sun , J. Du , J. Fan , X. Peng , Smart Mol. 2024, e20240005. 10.1002/smo.20240005 PMC12118193 40626272

[9] R. Klajn , Chem. Soc. Rev. 2013, 43, 148. 23979515 10.1039/c3cs60181a

[10] A. H. Gelebart , D. Jan Mulder , M. Varga , A. Konya , G. Vantomme , E. W. Meijer , R. L. B. Selinger , D. J. Broer , Nature 2017, 546, 632. 28658225 10.1038/nature22987 PMC5495175

[11] J. Gemen , J. R. Church , T.‐P. Ruoko , N. Durandin , M. J. Białek , M. Weißenfels , M. Feller , M. Kazes , M. Odaybat , V. A. Borin , R. Kalepu , Y. Diskin‐Posner , D. Oron , M. J. Fuchter , A. Priimagi , I. Schapiro , R. Klajn , Science 2023, 381, 1357. 37733864 10.1126/science.adh9059

[12] H.‐B. Cheng , S. Zhang , E. Bai , X. Cao , J. Wang , J. Qi , J. Liu , J. Zhao , L. Zhang , J. Yoon , Adv. Mater. 2022, 34, 2108289. 10.1002/adma.202108289 34866257

[13] S. Lin , K. G. Gutierrez‐Cuevas , X. Zhang , J. Guo , Q. Li , Adv. Funct. Mater. 2021, 31, 2007957.

[14] H. Chen , F. Yang , Q. Chen , J. Zheng , Adv. Mater. 2017, 29, 1606900. 10.1002/adma.201606900 28295677

[15] L. Kortekaas , W. R. Browne , Chem. Soc. Rev. 2019, 48, 3406. 31150035 10.1039/c9cs00203k

[16] D. Cao , Z. Liu , P. Verwilst , S. Koo , P. Jangjili , J. S. Kim , W. Lin , Chem. Rev. 2019, 119, 10403. 31314507 10.1021/acs.chemrev.9b00145

[17] L. Sansalone , J. Zhao , L. T. B. Nguyen , S. Gupta , D. L. Benson , M. Abe , G. C. R. Ellis‐Davies , Angew. Chem. Int. Ed. 2024, 63, e202315726. 10.1002/anie.202315726 PMC10947816 38329885

[18] H. Zhang , X. Li , Y. Lin , F. Gao , Z. Tang , P. Su , W. Zhang , Y. Xu , W. Weng , R. Boulatov , Nat. Commun. 2017, 8, 1147. 29079772 10.1038/s41467-017-01412-8 PMC5660084

[19] L. Su , W. Zhu , B. Liu , H.‐Q. Peng , ACS Mater. Lett. 2024, 6, 3533.

[20] Q. Song , K. Zhao , T. Xue , S. Zhao , D. Pei , J. Nie , Y. Chang , Macromolecules 2021, 54, 8314.

[21] H. Wu , F. Xu , G. Gao , X. Feng , Macromolecules 2021, 54, 5856.

[22] Y. Liu , Y. Lin , Y. Cao , A. Zhi , J. Chen , W. Li , B. Demir , D. J. Searles , A. K. Whittaker , A. Zhang , Chem. Commun. 2021, 57, 12780. 10.1039/d1cc05358b 34781324

[23] Y. Li , N. Busatto , P. J. Roth , Macromolecules 2021, 54, 3101.

[24] F. Wang , C. E. Diesendruck , Macromol. Rapid Commun. 2018, 39, 1700519. 10.1002/marc.201700519 29105907

[25] X. Zhu , Y. Jia , Y. Liu , J. Xu , H. He , S. Wang , Y. Shao , Y. Zhai , Y. Zhu , Angew. Chem. Int. Ed. 2024, 63, e202405962. 10.1002/anie.202405962 38644535

[26] Z. Lei , P. Wu , Nat. Commun. 2018, 9, 1134. 29555905 10.1038/s41467-018-03456-w PMC5859265

[27] D. Hoenders , S. Ludwanowski , C. Barner‐Kowollik , A. Walther , Angew. Chem. Int. Ed. 2024, 63, e202405582. 10.1002/anie.202405582 38640085

[28] H. Yang , B. Yuan , X. Zhang , O. A. Scherman , Acc. Chem. Res. 2014, 47, 2106. 24766328 10.1021/ar500105t

[29] Y.‐Y. Ren , B.‐Y. Deng , Z.‐H. Liao , Z.‐R. Zhou , C.‐H. Tung , L.‐Z. Wu , F. Wang , Adv. Mater. 2023, 35, 2307971. 10.1002/adma.202307971 37743568

[30] Y.‐M. Zhang , Y.‐H. Liu , Y. Liu , Adv. Mater. 2020, 32, 1806158.

[31] J. Liu , C. S. Y. Tan , Z. Yu , Y. Lan , C. Abell , O. A. Scherman , Adv. Mater. 2017, 29, 1604951. 10.1002/adma.201604951 28092128

[32] J. Li , D. Yim , W.‐D. Jang , J. Yoon , Chem. Soc. Rev. 2017, 46, 2437. 27711665 10.1039/c6cs00619a

[33] Y. Mi , J. Ma , W. Liang , C. Xiao , W. Wu , D. Zhou , J. Yao , W. Sun , J. Sun , G. Gao , X. Chen , J. J. Chruma , C. Yang , J. Am. Chem. Soc. 2021, 143, 1553. 33432813 10.1021/jacs.0c11833

[34] X. Ma , H. Tian , Acc. Chem. Res. 2014, 47, 1971. 24669851 10.1021/ar500033n

[35] C. Cardenas‐Daw , F. Gröhn , J. Am. Chem. Soc. 2015, 137, 8660. 26088975 10.1021/jacs.5b01357

[36] S. Li , Q. Zou , Y. Li , C. Yuan , R. Xing , X. Yan , J. Am. Chem. Soc. 2018, 140, 10794. 30102029 10.1021/jacs.8b04912

[37] C. He , F. Liang , L. Veeramuthu , C. Cho , J. Benas , Y. Tzeng , Y. Tseng , W. Chen , A. Rwei , C. Kuo , Adv. Sci. 2021, 8, 2102275. 10.1002/advs.202102275 PMC8564429 34519441

[38] Q. Wang , M. Cheng , J.‐L. Jiang , L.‐Y. Wang , Chin. Chem. Lett. 2017, 28, 793.

[39] X. Zhu , B. Liu , P. Cui , S. Kilina , W. Sun , Inorg. Chem. 2020, 59, 17096. 33170657 10.1021/acs.inorgchem.0c02366

[40] J. Zhang , H. Shen , X. Liu , X. Yang , S. L. Broman , H. Wang , Q. Li , J. W. Y. Lam , H. Zhang , M. Cacciarini , M. B. Nielsen , B. Z. Tang , Angew. Chem. Int. Ed. 2022, 61, e202208460. 10.1002/anie.202208460 35841180

[41] Z. Zhang , W. Wang , P. Jin , J. Xue , L. Sun , J. Huang , J. Zhang , H. Tian , Nat. Commun. 2019, 10, 4232. 31530814 10.1038/s41467-019-12302-6 PMC6748945

[42] X. Zhu , R. Liu , Y. Li , H. Huang , Q. Wang , D. Wang , X. Zhu , S. Liu , H. Zhu , Chem. Commun. 2014, 50, 12951. 10.1039/c4cc05913a 25220502

[43] B.‐Y. Deng , Z.‐R. Zhou , H.‐L. Xu , Z.‐H. Liao , C.‐H. Tung , L.‐Z. Wu , F. Wang , Small 2024, 20, 2311058. 10.1002/smll.202311058 38351656

[44] Q. Liu , M. Xie , X. Chang , S. Cao , C. Zou , W.‐F. Fu , C.‐M. Che , Y. Chen , W. Lu , Angew. Chem. Int. Ed. 2018, 57, 6279. 10.1002/anie.201803965 29655269

[45] Q. Wang , Q. Tan , S. Zhao , K. Zhang , J. Chen , M. Lan , Chem. Eng. J. 2023, 470, 144061.

[46] Z. Zhou , L. Zhang , L. Peng , Y. Li , X. Zhu , Y. Wu , Z. Qiu , G. He , M. Qin , H. Peng , Y. Fang , Aggregate 2024, e629.

[47] Z. Zheng , H. Hu , Z. Zhang , B. Liu , M. Li , D.‐H. Qu , H. Tian , W.‐H. Zhu , B. L. Feringa , Nat. Photonics 2022, 16, 226.

[48] H. Wu , Y. Chen , X. Dai , P. Li , J. F. Stoddart , Y. Liu , J. Am. Chem. Soc. 2019, 141, 6583. 30860369 10.1021/jacs.8b13675

[49] D. Kim , K. Jeong , J. E. Kwon , H. Park , S. Lee , S. Kim , S. Y. Park , Nat. Commun. 2019, 10, 3089. 31300649 10.1038/s41467-019-10986-4 PMC6626011

[50] R. Zhang , Y. Chen , Y. Liu , Angew. Chem. Int. Ed. 2023, 62, e202315749. 10.1002/anie.202315749 37971202

[51] Z. Li , X. Liu , G. Wang , B. Li , H. Chen , H. Li , Y. Zhao , Nat. Commun. 2021, 12, 1363. 33649315 10.1038/s41467-021-21677-4 PMC7921134

[52] H.‐Q. Zheng , Y. Yang , Z. Wang , D. Yang , G. Qian , Y. Cui , Adv. Mater. 2023, 35, 2300177. 10.1002/adma.202300177 36893771

[53] Y.‐X. Hu , X. Hao , D. Wang , Z.‐C. Zhang , H. Sun , X.‐D. Xu , X. Xie , X. Shi , H. Peng , H.‐B. Yang , L. Xu , Angew. Chem. Int. Ed. 2024, 63, e202315061. 10.1002/anie.202315061 37966368

[54] M. Li , J. Guo , C. Zhang , Y. Che , Y. Yi , B. Liu , Angew. Chem. Int. Ed. 2023, 62, e202309914. 10.1002/anie.202309914 37837298

[55] H. Luo , Z. Wang , F. Yu , Z. Zhou , J. Wang , D. Chen , Q. Feng , X. Cao , Adv. Funct. Mater. 2024, 34, 2315592.

[56] E. Fuentes , M. Gerth , J. A. Berrocal , C. Matera , P. Gorostiza , I. K. Voets , S. Pujals , L. Albertazzi , J. Am. Chem. Soc. 2020, 142, 10069. 32395995 10.1021/jacs.0c02067 PMC7497294

[57] S. Yan , K. Hu , S. Chen , T. Li , W. Zhang , J. Yin , X. Jiang , Nat. Commun. 2022, 13, 7434. 36460720 10.1038/s41467-022-35271-9 PMC9718802

[58] Y. Nagai , K. Ishiba , R. Yamamoto , T. Yamada , M. Morikawa , N. Kimizuka , Angew. Chem. Int. Ed. 2021, 60, 6333. 10.1002/anie.202013650 33350044

[59] C. Ni , D. Chen , Y. Yin , X. Wen , X. Chen , C. Yang , G. Chen , Z. Sun , J. Wen , Y. Jiao , C. Wang , N. Wang , X. Kong , S. Deng , Y. Shen , R. Xiao , X. Jin , J. Li , X. Kong , Q. Zhao , T. Xie , Nature 2023, 622, 748. 37704734 10.1038/s41586-023-06520-8

[60] J. Hou , G. Long , W. Zhao , G. Zhou , D. Liu , D. J. Broer , B. L. Feringa , J. Chen , J. Am. Chem. Soc. 2022, 144, 6851. 35380815 10.1021/jacs.2c01060 PMC9026258

[61] N. J. Oldenhuis , K. P. Qin , S. Wang , H.‐Z. Ye , E. A. Alt , A. P. Willard , T. Van Voorhis , S. L. Craig , J. A. Johnson , Angew. Chem. Int. Ed. 2020, 59, 2784. 10.1002/anie.201913297 PMC7187918 31742840

[62] C. Li , A. Iscen , L. C. Palmer , G. C. Schatz , S. I. Stupp , J. Am. Chem. Soc. 2020, 142, 8447. 32330027 10.1021/jacs.0c02201

[63] V. Udyavara Nagaraj , T. Juhász , M. Quemé‐Peña , I. Cs. Szigyártó , D. Bogdán , A. Wacha , J. Mihály , L. Románszki , Z. Varga , J. Andréasson , I. Mándity , T. Beke‐Somfai , ACS Appl. Mater. Interfaces 2022, 14, 55320. 36473125 10.1021/acsami.2c11946 PMC9782321

[64] Y. Shan , Q. Zhang , J. Sheng , M. C. A. Stuart , D.‐H. Qu , B. L. Feringa , Angew. Chem. Int. Ed. 2023, 62, e202310582. 10.1002/anie.202310582 37681477

[65] Y. Xiang , H. Mao , S. Tong , C. Liu , R. Yan , L. Zhao , L. Zhu , C. Bao , ACS Nano 2023, 17, 5536. 36892586 10.1021/acsnano.2c10896

[66] K. Guo , X. Yang , C. Zhou , C. Li , Nat. Commun. 2024, 15, 1694. 38402204 10.1038/s41467-024-46100-6 PMC10894256

[67] C. Li , A. Iscen , H. Sai , K. Sato , N. A. Sather , S. M. Chin , Z. Álvarez , L. C. Palmer , G. C. Schatz , S. I. Stupp , Nat. Mater. 2020, 19, 900. 32572204 10.1038/s41563-020-0707-7

[68] Y.‐L. Lin , S. Zheng , C.‐C. Chang , L.‐R. Lee , J.‐T. Chen , Nat. Commun. 2024, 15, 916. 38296994 10.1038/s41467-024-45188-0 PMC10831044

[69] A. Legrand , L.‐H. Liu , P. Royla , T. Aoyama , G. A. Craig , A. Carné‐Sánchez , K. Urayama , J. J. Weigand , C.‐H. Lin , S. Furukawa , J. Am. Chem. Soc. 2021, 143, 3562. 33646776 10.1021/jacs.1c00108

[70] Y.‐F. Chen , C.‐L. Hsieh , P.‐Y. Lin , Y.‐C. Liu , M.‐J. Lee , L.‐R. Lee , S. Zheng , Y.‐L. Lin , Y.‐L. Huang , J.‐T. Chen , Small 2024, 20, 2305317. 10.1002/smll.202305317 37670223

[71] J. Lee , K. H. Ku , J. Kim , Y. J. Lee , S. G. Jang , B. J. Kim , J. Am. Chem. Soc. 2019, 141, 15348. 31433168 10.1021/jacs.9b07755

[72] W. R. Berg , J. F. Berengut , C. Bai , L. Wimberger , L. K. Lee , F. J. Rizzuto , Angew. Chem. Int. Ed. 2023, 62, e202314458. 10.1002/anie.202314458 37903739

[73] X. Zhu , Z. Wang , Y. Jia , F. Yang , Y. Zhang , S. Zhao , X. He , J. Mater. Chem. C 2023, 11, 11671.

[74] Y.‐H. Yang , Y. Qin , Y. Zhang , L. Zhang , Aggregate 2023, 4, e268.

[75] L. Zhou , P. Retailleau , M. Morel , S. Rudiuk , D. Baigl , J. Am. Chem. Soc. 2019, 141, 9321. 31117648 10.1021/jacs.9b02836

[76] J. S. Valera , H. Arima , C. Naranjo , T. Saito , N. Suda , R. Gómez , S. Yagai , L. Sánchez , Angew. Chem. Int. Ed. 2022, 61, e202114290. 10.1002/anie.202114290 PMC9299728 34822210

[77] N. Sasaki , M. F. J. Mabesoone , J. Kikkawa , T. Fukui , N. Shioya , T. Shimoaka , T. Hasegawa , H. Takagi , R. Haruki , N. Shimizu , S. Adachi , E. W. Meijer , M. Takeuchi , K. Sugiyasu , Nat. Commun. 2020, 11, 3578. 32681045 10.1038/s41467-020-17356-5 PMC7368029

[78] A. Abdollahi , A. Herizchi , H. Roghani‐Mamaqani , H. Alidaei‐Sharif , Carbohydr. Polym. 2020, 230, 115603. 31887950 10.1016/j.carbpol.2019.115603

[79] J. Seo , J. F. Joung , S. Park , Y. J. Son , J. Noh , J.‐M. Kim , Nat. Commun. 2020, 11, 6260. 33288757 10.1038/s41467-020-20172-6 PMC7721704

[80] O. Vybornyi , S.‐X. Liu , R. Häner , Angew. Chem. Int. Ed. 2021, 60, 25872. 10.1002/anie.202108745 PMC9298031 34529324

[81] J. Kim , H. Yun , Y. J. Lee , J. Lee , S.‐H. Kim , K. H. Ku , B. J. Kim , J. Am. Chem. Soc. 2021, 143, 13333. 34379395 10.1021/jacs.1c06377

[82] X.‐M. Chen , W.‐J. Feng , H. K. Bisoyi , S. Zhang , X. Chen , H. Yang , Q. Li , Nat. Commun. 2022, 13, 3216. 35680948 10.1038/s41467-022-30969-2 PMC9184535

[83] Y. Xue , S. Jiang , H. Zhong , Z. Chen , F. Wang , Angew. Chem. Int. Ed. 2022, 61, e202110766. 10.1002/anie.202110766 34714571

[84] M. Zeng , W. Wang , S. Zhang , Z. Gao , Y. Yan , Y. Liu , Y. Qi , X. Yan , W. Zhao , X. Zhang , N. Guo , H. Li , H. Li , G. Xie , Y. Tao , R. Chen , W. Huang , Nat. Commun. 2024, 15, 3053. 38594234 10.1038/s41467-024-47240-5 PMC11004163

[85] T. Miao , X. Cheng , H. Ma , Z. He , Z. Zhang , N. Zhou , W. Zhang , X. Zhu , Angew. Chem. Int. Ed. 2021, 60, 18566. 10.1002/anie.202107992 34156135

[86] L. Wang , L. Yin , W. Zhang , X. Zhu , M. Fujiki , J. Am. Chem. Soc. 2017, 139, 13218. 28846842 10.1021/jacs.7b07626

[87] C. Kulkarni , R. H. N. Curvers , G. Vantomme , D. J. Broer , A. R. A. Palmans , S. C. J. Meskers , E. W. Meijer , Adv. Mater. 2021, 33, 2005720. 33270297 10.1002/adma.202005720 PMC11468155

[88] Y. Jiang , C. Zhang , R. Wang , Y. Lei , W. Dai , M. Liu , H. Wu , Y. Tao , X. Huang , Adv. Opt. Mater. 2024, 12, 2302482.

[89] S. Huang , Y. Chen , S. Ma , H. Yu , Angew. Chem. Int. Ed. 2018, 57, 12524. 10.1002/anie.201807379 30062805

[90] S. Li , J. Wang , M. Tian , X. Meng , J. Wang , J. Guo , Angew. Chem. Int. Ed. 2024, 63, e202405615. 10.1002/anie.202405615 38856204

[91] X. Cheng , T. Miao , Y. Ma , X. Zhu , W. Zhang , X. Zhu , Angew. Chem. Int. Ed. 2021, 60, 24430. 10.1002/anie.202109084 34505335

[92] H. Jiang , Y. Jiang , J. Han , L. Zhang , M. Liu , Angew. Chem. Int. Ed. 2019, 58, 785. 10.1002/anie.201811060 30426680

[93] F. Xu , S. Crespi , G. Pacella , Y. Fu , M. C. A. Stuart , Q. Zhang , G. Portale , B. L. Feringa , J. Am. Chem. Soc. 2022, 144, 6019. 35341243 10.1021/jacs.2c01063 PMC8991000

[94] W. Yuan , L. Chen , C. Yuan , Z. Zhang , X. Chen , X. Zhang , J. Guo , C. Qian , Z. Zhao , Y. Zhao , Nat. Commun. 2023, 14, 8022. 38049414 10.1038/s41467-023-43830-x PMC10696047

[95] Y. Guo , X. Cheng , Z. He , Z. Zhou , T. Miao , W. Zhang , Angew. Chem. Int. Ed. 2023, 62, e202312259. 10.1002/anie.202312259 37738071

[96] Y. Zhang , M. Qin , C. Xing , C. Zhao , X. Dou , C. Feng , ACS Nano 2020, 14, 17151. 33202135 10.1021/acsnano.0c06938

[97] L. Huang , D. Qing , S. Zhao , X. Wu , K. Yang , X. Ren , X. Zheng , M. Lan , J. Ye , L. Zeng , G. Niu , Chem. Eng. J. 2022, 430, 132638.

[98] D. Wang , T. Zhang , B. Wu , C. Ye , Z. Wei , Z. Cao , G. Wang , Macromolecules 2019, 52, 7130.

[99] P. Donthamsetti , D. B. Konrad , B. Hetzler , Z. Fu , D. Trauner , E. Y. Isacoff , J. Am. Chem. Soc. 2021, 143, 8951. 34115935 10.1021/jacs.1c02586 PMC8227462

[100] L. Li , H. Sun , M. Li , Y. Yang , T. P. Russell , S. Shi , Angew. Chem. Int. Ed. 2021, 60, 17394. 10.1002/anie.202105500 34046998

[101] R.‐J. Li , Julian J. Holstein , W. G. Hiller , J. Andréasson , G. H. Clever , J. Am. Chem. Soc. 2019, 141, 2097. 30620873 10.1021/jacs.8b11872

[102] F. Zhu , X. Chen , Y. Ge , M. Li , N. Li , J. Zhou , W. Chen , Adv. Funct. Mater. 2023, 2312155.

[103] T. Wang , Y. Qi , E. Miyako , A. Bianco , C. Ménard‐Moyon , Small 2024, 20, 2307337. 10.1002/smll.202307337 38152926

[104] H. Lu , H. Ye , L. You , J. Am. Chem. Soc. 2024, 146, 11392.

[105] J. Zhang , Y. Fu , H.‐H. Han , Y. Zang , J. Li , X.‐P. He , B. L. Feringa , H. Tian , Nat. Commun. 2017, 8, 987. 29042558 10.1038/s41467-017-01137-8 PMC5715093

[106] Y. Gao , S.‐C. Yang , M.‐H. Zhu , X.‐D. Zhu , X. Luan , X.‐L. Liu , X. Lai , Y. Yuan , Q. Lu , P. Sun , J. F. Lovell , H.‐Z. Chen , C. Fang , Small 2021, 17, 2100789. 10.1002/smll.202100789 34142432

[107] S. Cao , L. C. da Silva , K. Landfester , Angew. Chem. Int. Ed. 2022, 61, e202205266. 10.1002/anie.202205266 PMC9542181 35759257

[108] K. M. Lakshmi , J. V. Rival , P. Sreeraj , S. R. Nambiar , C. Jeyabharathi , Nonappa , E. S. Shibu , Small 2023, 19, 2207119. 10.1002/smll.202207119 36683222

[109] Y. Wu , Y. Yang , Y. Zhang , L. Dai , W. Dong , H. He , H. Li , Z. Nie , Y. Sang , Angew. Chem. Int. Ed. 2024, 63, e202313406. 10.1002/anie.202313406 37801444

[110] Z. Xiao , S. Duan , P. Xu , J. Cui , H. Zhang , W. Wang , ACS Nano 2020, 14, 8658. 32530617 10.1021/acsnano.0c03022

[111] Y. Mu , W. Duan , K. Y. Hsu , Z. Wang , W. Xu , Y. Wang , ACS Appl. Mater. Interfaces 2022, 14, 57113. 36512379 10.1021/acsami.2c14551

[112] M. Yang , Y. Xu , X. Zhang , H. K. Bisoyi , P. Xue , Y. Yang , X. Yang , C. Valenzuela , Y. Chen , L. Wang , W. Feng , Q. Li , Adv. Funct. Mater. 2022, 32, 2201884.

[113] P. Feng , X. Du , J. Guo , K. Wang , B. Song , ACS Appl. Mater. Interfaces 2021, 13, 8985. 33583177 10.1021/acsami.0c22340

[114] S. Palagi , A. G. Mark , S. Y. Reigh , K. Melde , T. Qiu , H. Zeng , C. Parmeggiani , D. Martella , A. Sanchez‐Castillo , N. Kapernaum , F. Giesselmann , D. S. Wiersma , E. Lauga , P. Fischer , Nat. Mater. 2016, 15, 647. 26878315 10.1038/nmat4569

[115] C. Gao , Y. Wang , Z. Ye , Z. Lin , X. Ma , Q. He , Adv. Mater. 2021, 33, 2000512. 10.1002/adma.202000512 32578282

[116] O. M. Wani , H. Zeng , A. Priimagi , Nat. Commun. 2017, 8, 15546. 28534872 10.1038/ncomms15546 PMC5457518

[117] M. Urso , M. Ussia , X. Peng , C. M. Oral , M. Pumera , Nat. Commun. 2023, 14, 6969. 37914692 10.1038/s41467-023-42674-9 PMC10620202

[118] X. Peng , M. Urso , M. Ussia , M. Pumera , ACS Nano 2022, 16, 7615. 35451832 10.1021/acsnano.1c11136

[119] Z. Ye , Y. Wang , S. Liu , D. Xu , W. Wang , X. Ma , J. Am. Chem. Soc. 2021, 143, 15063. 34499495 10.1021/jacs.1c04836

[120] J. Yao , W. Wu , C. Xiao , D. Su , Z. Zhong , T. Mori , C. Yang , Nat. Commun. 2021, 12, 2600. 33972556 10.1038/s41467-021-22880-z PMC8110520

[121] Y. Liu , Q. Zhang , S. Crespi , S. Chen , X.‐K. Zhang , T.‐Y. Xu , C.‐S. Ma , S.‐W. Zhou , Z.‐T. Shi , H. Tian , B. L. Feringa , D.‐H. Qu , Angew. Chem. Int. Ed. 2021, 60, 16129. 10.1002/anie.202104285 PMC8361693 33955650

